# Mebendazole plus lomustine or temozolomide in patients with recurrent glioblastoma: A randomised open-label phase II trial

**DOI:** 10.1016/j.eclinm.2022.101449

**Published:** 2022-05-27

**Authors:** Vijay M. Patil, Nandini Menon, Abhishek Chatterjee, Raees Tonse, Amit Choudhari, Abhishek Mahajan, Ameya D. Puranik, Sridhar Epari, Monica Jadhav, Shruti Pathak, Zoya Peelay, Rutuja Walavalkar, Hemanth K. Muthuluri, Madala Ravi Krishna, Arun Chandrasekharan, Nikhil Pande, Tejpal Gupta, Shripad Banavali, Rakesh Jalali

**Affiliations:** a Department of Medical Oncology, Tata Memorial Center, Mumbai, India; b Department of Radiation Oncology, Tata Memorial Center, Mumbai, India; c Department of Radiology, Tata Memorial Center, Mumbai, India; d Department of Nuclear Medicine, Tata Memorial Center, Mumbai, India; e Department of Pathology, Tata Memorial Center, Mumbai, India; f Homi Bhabha National Institute (HBNI), Mumbai, India

**Keywords:** Mebendazole, Glioblastoma, Recurrent, Salvage, CCNU

## Abstract

**Background:**

Recurrent glioblastoma (GBM) has dismal outcomes and limited treatment options. Mebendazole (MBZ) has activity in glioma both *in-vivo* and *in-vitro*, and is well tolerated in combination with lomustine (CCNU) and temozolomide (TMZ). In this study, we sought to determine whether the addition of MBZ to CCNU or TMZ would improve overall survival (OS) in recurrent GBM.

**Methods:**

In this phase II randomized open-label trial, adult patients with ECOG PS 0–3, with recurrent GBM who were not eligible for re-radiation, were randomized 1:1 to the CCNU-MBZ and TMZ-MBZ arms. CCNU was administered at 110 mg/m^2^ every 6 weeks with MBZ 800 mg thrice daily and TMZ was administered at 200 mg/m^2^ once daily on days 1–5 of a 28 days cycle with MBZ 1600 mg thrice daily. The primary endpoint was OS at 9 months. A 9-month OS of 55% or more in any arm was hypothesized to warrant further evaluation and a value below 35% was too low to warrant further investigation. OS was analyzed using intention to treat (ITT) and per-protocol (PP) analyses. Per-protocol analysis was used for safety analysis. Clinical Trials Registry-India number, CTRI/2018/01/011542.

**Findings:**

Participants were recruited from 14^th^ March 2019 to 18^th^ June 2021, 44 patients were randomised on each arm. At 17.4 months, 68 events for OS analysis had occurred, 33 in the TMZ-MBZ and 35 in the CCNU-MBZ arm. The 9-month OS was 36.6% (95% CI 22.3–51.0) and 45% (95% CI 29.6–59.2) in the TMZ-MBZ and CCNU-MBZ arms respectively, in the ITT population. ECOG PS was the only independent prognostic factor impacting OS (HR-0.48, 95% CI 0.27–0.85; *P* = 0.012). Grade 3–5 adverse events were seen in 8 (18.6%; *n* = 43) and 4 (9.5%; *n* = 42) patients in the TMZ-MBZ and CCNU-MBZ arms respectively. There were no treatment related deaths.

**Interpretation:**

The addition of MBZ to TMZ or CCNU failed to achieve the pre-set benchmark of 55% 9-month OS. This was probably due to 28.6% of patients having poor PS of 2–3.

**Funding:**

Brain Tumor Foundation (BTF) of India, Indian Cooperative Oncology Network (ICON), and India Cancer Research Consortium (ICRC) under ICMR (Indian Council of Medical Research).

**Research in context**

**Evidence before this study**

A PubMed literature search was performed on 1 April 2022, with the MeSH terms, “Mebendazole” and “Glioma” without any filters or language restrictions. Only 17 articles were found, of which 7 were relevant. The data suggests that mebendazole (MBZ) has *in vivo* and *in-vitro* activity against glioma cell lines. We conducted a phase I//II study, to identify the maximum tolerable dose of mebendazole in combination with various salvage treatment options used in recurrent glioma (in phase I), and to study its efficacy in terms of 9-month overall survival (in phase II). Results of the phase I part of the study were published in May 2020. Subsequently, Gallia et al. published results of their phase I study on mebendazole (MBZ) with adjuvant temozolomide in patients with newly diagnosed high-grade gliomas, which demonstrated the safety of MBZ at doses up to 200 mg/kg with acceptable toxicity. Here, we report the efficacy outcomes of MBZ in combination with Lomustine (CCNU) or Temozolomide (TMZ), in terms of 9-month overall survival (OS) in adults with recurrent glioma who are not eligible for re-irradiation, which is a subset of patients with few treatment options and dismal outcomes.

**Added value of this study**

This is the first in-human phase II data for the efficacy of Mebendazole in this setting. In this study, the 9-month OS was 36.6% (95%CI 22.3–51.0) and 45.0% (95%CI 29.6–59.2) in the TMZ-MBZ and CCNU-MBZ arms respectively (on intention to treat analysis), which did not meet the pre-specified benchmark of 55%. In the previous BELOB trial, 9-month OS in the CCNU + Bevacizumab group was 63% (95% CI 49–75). However, it is important to note that 28.6% of patients in our study had an ECOG PS (Eastern Cooperative Oncology Group Performance Status) 2–3, which is higher than the proportion of patients with ECOG PS 2 or 3 in other studies on recurrent glioblastoma; such as the BELOB study (PS 2, 11.5%; PS 3, 0%) and the EORTC 26,101 study (PS 2, 10.2%; PS 3, 0%). In the post-hoc analyses, patients with ECOG PS 2–3 had an inferior OS rate compared with those who had an ECOG PS of 0–1.

**Implications of all evidence available**

The available evidence so far establishes the safety and the dose of MBZ that can be further evaluated in glioblastoma. The addition of MBZ to TMZ or CCNU failed to achieve the pre-set benchmark of 55% 9-month OS.

Alt-text: Unlabelled box

## Introduction

Glioblastoma (GBM) is an uncommon tumor^1^ and is associated with a dismal prognosis. Despite maximal safe resection, external beam radiation, concurrent, and adjuvant temozolomide, the median progression-free survival (PFS) in the European Organization for Research and Treatment of Cancer–National Cancer Institute of Canada randomized trial was only 6.9 months (95% CI 5.8–8.2).^2^ The median overall survival (OS) in this trial was 14.6 months (95% CI 13.2–16.8) and 12 months OS was 61.1% (95% CI 55.4–66.7).^2^ The small delta between median PFS and OS suggests the very limited impact of second-line treatments. In a recent phase 3 trial, the addition of tumor-treating fields therapy (TTF) demonstrated further improvement in PFS and OS, but the gains remain very modest. The median PFS in patients treated with TTF-temozolomide was 6.7 months versus 4.0 months in those treated with temozolomide-alone (HR, 0.63; 95% CI, 0.52–0.76; *P* < 0.001) while the median overall survival was 20.9 months and 16.0 months in the TTF-temozolomide and temozolomide-alone groups respectively; (HR, 0.63; 95% CI, 0.53–0.76; *P* < 0.001).^3^ The improvement in 12-month OS was from 65% (59–72) to 73% (69–77). Available options for salvage include re-resection, re-irradiation, systemic therapy, and tumor-treating field therapy.^4^ Unfortunately, because of the diffuse nature of relapse, few patients with recurrent GBM are eligible for local therapeutic strategies, and hence the majority are offered either systemic therapy or best supportive care.^5^These systemic therapeutic options remain non-categorical because of poor rates of salvage and include, amongst others, lomustine (CCNU), temozolomide (TMZ), PCV (Procarbazine, CCNU & vincristine), bevacizumab, regorafenib or a combination of these drugs.^6^

In large centres, clinical trials remain the preferred default strategy for these patients. Unfortunately, all of these approaches have rather similar, almost uniformly poor outcomes and none has demonstrated categorical superiority.^7^, ^8^, ^9^, ^10^In light of the unsatisfactory results with these agents, with a median OS after relapse of only 6.0–8.0 months and a 9 month OS of 50%, there is an urgent need for newer approaches.

Mebendazole (Methyl 5-benzoyl-2-benzimidazole carbamate) is an anti-helminthic drug with *in-vivo* and *in-vitro* data demonstrating effectiveness against glioma models.^11^^,^^12^ Mebendazole in combination with TMZ has demonstrated safety (at doses up to 200 mg/kg) and promising activity in newly diagnosed high-grade gliomas.^12^ It also has activity in temozolomide-resistant glioma cell lines.^11^ In view of the above-mentioned activity, lipophilic nature, ability to cross the blood-brain barrier,^13^ ease of administration (oral), long safety experience from using mebendazole in various helminthic infestations^14^; we initially conducted a phase I combinatorial study with CCNU, and separately with temozolomide alone or temozolomide plus radiotherapy, to identify potential toxicities and define the recommended phase II dose for a larger randomized phase II trial. In that phase I study, mebendazole was well tolerated in all of the arms, with no unanticipated serious adverse events.^15^ The recommended phase II dose of mebendazole was determined to be 1600 mg thrice daily with temozolomide and temozolomide-radiation combination, and 800 mg thrice daily with CCNU. The phase I trial was expanded to an initial signal-seeking phase II trial, results of which are reported herein.

## Methods

### Study design

This was a multi-arm, open label, phase I trial with built-in expansion to a randomized phase II component. Phase I has been completed and published. The study was conducted at the Tata Memorial Center, a tertiary cancer center in Mumbai, India. The study protocol was approved by the Institutional Ethics Committee (IEC). The study was conducted in accordance with the principles of the Declaration of Helsinki and the Good Clinical Practice guidelines of the International Conference on Harmonization. It was monitored by an independent data monitoring safety board.

The reirradiation arm is still recruiting patients and its results will be reported separately once enrollment is completed and data are analyzed. Here, we report the results of the salvage chemotherapy plus mebendazole arms of the phase II component of the trial, in accordance with the CONSORT reporting guidelines.

### Participants

Adult patients (age ≥ 18 years) with recurrent glioblastoma, an Eastern Cooperative Oncology Group performance status (ECOG PS) score of 0- 3, and normal organ and bone marrow functions were eligible for the study. Patients were assessed for eligibility for salvage re-irradiation after discussion in a multidisciplinary tumor board. Patients with a good performance status (Karnofsky score ≥60) at re-irradiation, prolonged time-interval from the first course of irradiation (at least 2-years), and recurrence confined to a single site in the supratentorial brain (moderate volume disease) were eligible for re-irradiation. Debulking surgery though preferred was not mandated at the time of recurrence/progression prior to re-irradiation. Patients who had received any benzimidazole in the preceding 3 months, those with recurrence within 3 months of discontinuing temozolomide, patients with any uncontrolled comorbidities, pregnant or lactating females, and patients with a history of previous life-threatening complications with temozolomide were excluded. The complete set of inclusion and exclusion criteria is provided in the study protocol in the supplementary appendix. Written informed consent was obtained from all patients prior to participation.

### Randomization and masking

Patients not eligible for reirradiation were randomly assigned to either temozolomide-mebendazole (TMZ-MBZ) or CCNU-mebendazole (CCNU-MBZ) in a 1:1 distribution. A simple randomization sheet was generated by an independent statistician. The study team did not have access to the sheet. Central randomization was performed by an independent person. The study team would send the patient details via e-mail to the independent person who did the randomization, and the randomization arm was conveyed to the study team via e-mail.

### Procedures

Patients enrolled in the re-irradiation arm received TMZ 75 mg/m^2^ daily with mebendazole (1600 mg thrice a day) during radiotherapy followed by TMZ 200 mg/m^2^ on days 1 to 5 of a 28-day cycle with mebendazole 1600 mg thrice daily, for a maximum of 12 cycles.

Patients in the TMZ-MBZ arm received Temozolomide 200 mg/m^2^ once daily from days 1 to 5, administered 2 hours post breakfast, with a concurrent 5 HT-3 inhibitor. Cycles were repeated every 28 days and a maximum of 12 cycles were planned. Oral mebendazole was dosed at 1600 mg thrice daily (a total dose of 4800 mg daily) till progression. In the CCNU-MBZ arm, CCNU was dosed at 110 mg/m^2^ once on day 1 of each 42-day cycle for a maximum of 6 cycles, administered with a concurrent 5HT-3 inhibitor; mebendazole was dosed at 800 mg thrice daily (a total dose of 2400 mg daily) till progression. Generic mebendazole isoform B in the form of chewable tablets (Tablet Mebex 100 mg, Cipla Limited) was used in this study for all patients. The drugs for this study were sourced through the hospital pharmacy which follows a strict quality control protocol as per the national and international guidelines.

Patients in both arms were assessed at baseline, on day 1 of each cycle, and 1 month after treatment completion (i.e., after cycle 12 in the TMZ-MBZ arm and cycle 6 in CCNU -MBZ arm) and 2 monthly thereafter till disease progression.

Response assessment was performed every 6 cycles in the TMZ-MBZ arm and every 3 cycles in the CCNU-MBZ arm, according to the Response Assessment in Neuro-Oncology (RANO) criteria. It was also done in the case of suspected clinical progression. Quality of life (QoL) assessments were performed in each arm at baseline, 3 months (+/- 15 days), and at 6 months (+/- 1 month). European Organization for Research and Treatment of Cancer (EORTC) QLQ C-30 with Brain (QLQ-BN20) questionnaires were used for QoL assessments.

### Outcomes

The primary outcome for the phase II part of this study was the 9-month overall survival (OS). Overall survival (OS) was calculated from the date of randomization to death. The secondary outcomes were progression-free survival (PFS), toxicity, and quality of life. Progression-free survival (PFS) was calculated from the date of randomization to the date of disease progression. Adverse events were recorded at each visit as per Common Terminology Criteria for Adverse Events (CTCAE) version 4.03.

### Statistical analysis

An A’ Hern one-stage design was used to calculate the sample size needed for each of the treatment arms. With P0 set at 35% (i.e., a true overall survival at 9 months of 35% was judged to be too low to warrant further investigation) and P1 set at 55% (i.e., a true overall survival at 9 months of 55% was judged sufficient to warrant further evaluation) selected to reflect the BELOB trial,^9^ and with α of 0·10 and β of 0·10, a total of 44 patients were needed per group. On the basis of these assumptions, if 20 of 44 or more patients were still alive at 9 months in any arm then that arm would warrant further investigation in clinical studies.

The OS and PFS were analyzed using intention to treat (ITT) as well as per-protocol (PP) analyses. OS and PFS were estimated using the Kaplan Meier method and median follow-up was estimated using the reverse Kaplan Meier method. The 9-month OS and median estimates with 95% confidence intervals (CI) were estimated for both arms, using the Brookmeyer and Crowley method. Data were censored for analysis on 13^th^ December 2021. The assumptions for proportional hazards were met and COX regression analysis was performed to identify factors impacting OS. Hazard ratios were calculated with Efron's method of tie handling. To circumvent the impact of the inclusion of ECOG PS 2–3 patients, a post hoc analysis of OS with ITT was performed with the patient population restricted to ECOG PS 0–1. RStudio version 1.0.136 (RStudio Team (2016), RStudio: Integrated Development for R. RStudio, Inc., Boston, MA URL http://www.rstudio.com/) and IBM SPSS version 20.0 statistics for Windows, (Armonk, NY: IBM Corp) were used for analysis. The study was registered prospectively with the Clinical Trials Registry- India (CTRI) [CTRI/2018/01/011542].

### Role of funding source

The funding agency had no role in the study design, the collection, analysis, and interpretation of data; the writing of the report; and in the decision to submit the paper for publication. All authors had access to the data set and decided to submit the manuscript for publication.

## Results

**Figure 1:**
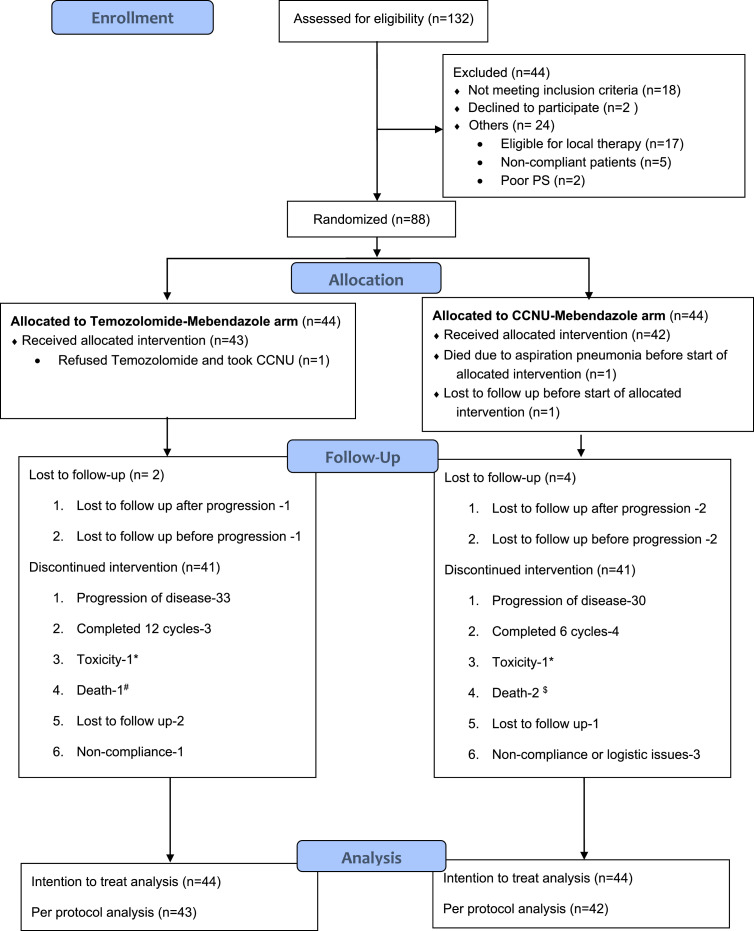
Consort diagram. *One patient discontinued TMZ due to deranged liver function tests and one patient discontinued CCNU due to myelosuppression. #-One patient died after the 1st cycle with symptoms suggestive of clinical progression. $-One patient before the start of any therapy due to aspiration pneumonia and one had seizures and died after the first cycle.

Eighty-eight patients were recruited from 14^th^ March 2019 to 18^th^ June 2021, 44 each in the TMZ-MBZ and CCNU-MBZ arms. Forty- four patients in each arm were included for the intention-to-treat analysis, while 43 patients in the TMZ-MBZ arm and 42 patients in the CCNU-MBZ were included for the per-protocol analysis. The CONSORT diagram is shown in Figure 1.

Key baseline characteristics include a relatively younger median age of around 41 years, commensurate with the epidemiologic nature of GBM in India, which corresponds to the high incidence of IDH mutations (36%), male preponderance (73–75%) and low rates of MGMT test availability (less than 50%). The baseline characteristics are shown in Table 1. All patients had undergone previous surgery and radiotherapy. All except 4 patients (2 in each arm) had also received concurrent and adjuvant TMZ.

**Table 1:** Baseline characteristics.

Characteristic	TMZ-MBZ arm (*n* = 44)	CCNU-MBZ arm (*n* = 44)	P-value
**Age (in years)**			
Median (Range)	40.5 (19–64)	41 (18–62)	
Elderly-no (%)	3 (6.8)	1 (2.3)	0.62
**Gender-no. (%)**			>0.99
Male	33 (75)	32 (72.7)	
Female	11 (25)	12 (27.3)	
**ECOG performance status-no. (%)**			>0.99
0–1	33 (75)	32 (72.7)	
2–3	11 (25)	12 (27.3)	
**Comorbidities-no. (%)**			
Hypertension	–	3 (6.8)	0.24
Diabetes	5 (11.4)	6 (13.6)	>0.99
**MGMT status-no. (%)**			0.91
Methylated	7 (15.9)	8 (18.2)	
Unmethylated	12 (27.3)	13 (29.5)	
Uninterpretable	1 (2.3)	2 (4.5)	
Not performed	24 (54.5)	21 (47.7)	
**IDH mutation status-no. (%)**			0.61
Negative	22 (50)	18 (40.9)	
Uninterpretable	–	1 (2.3)	
Positive	16 (36.4)	16 (36.4)	
Not performed	6 (13.6)	9 (20.5)	
**Steroid use-no. (%)**			0.66
Yes	17 (38.6)	14 (31.8)	
No	27 (61.4)	30 (68.2)	
**Immediate previous treatment intent-no. (%)**			>0.99
Curative	44 (100)	43 (97.7)	
Palliative	–	1 (2.3)	
**Previous cumulative exposure to temozolomide-no. (%)**			0.096
<=6 cycles*	23 (52.3)	18 (40.9)	
7–12 cycles	21 (47.7)	22 (50)	
>12 cycles	–	4 (9.1)	

ECOG PS: - Eastern Cooperative Oncology Group performance status, IDH - Presence of Isocitrate dehydrogenase 1 and 2 mutations, MGMT- Methylation of the O (6)-Methylguanine-DNA methyltransferase. Elderly was defined as age 60 years or more. TMZ-Temozolomide, MBZ-Mebendazole, CCNU-Lomustine.

* In the TMZ-MBZ arm 8 patients had received less than 6 cycles of TMZ, of these 2 patients did not receive any prior TMZ, 3 patients had treatment failure within 6 months & 3 patients chose to discontinue TMZ due to logistic/personal reasons. In the CCNU-MBZ arm, 7 patients had received less than 6 cycles of prior TMZ, 2 patients did not receive any prior TMZ, 3 had treatment failure within 6 months and 2 patients chose to discontinue TMZ due to logistic/personal reasons.

### Primary outcome - overall survival

**Figure 2:**
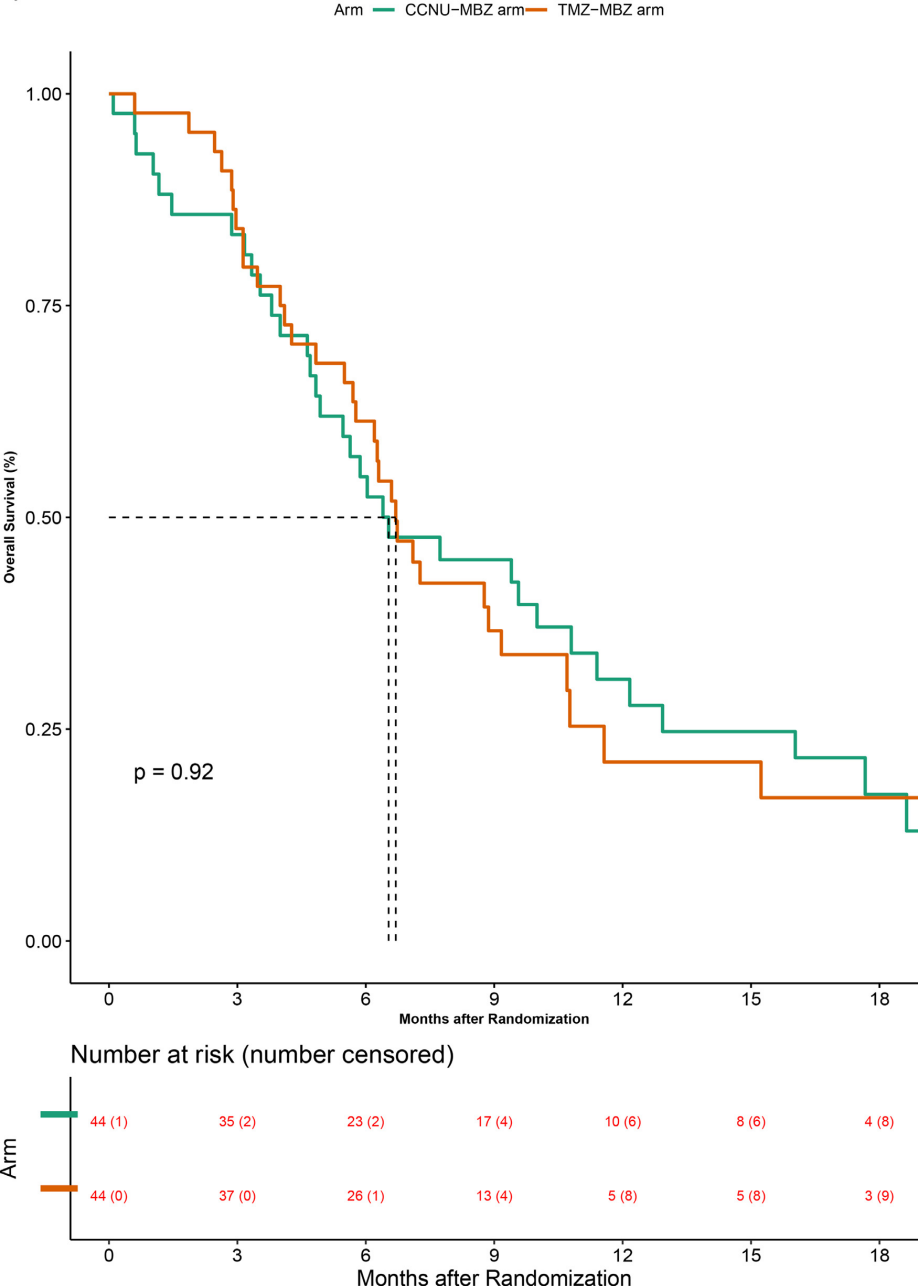
Overall survival in the Intention to treat population. The orange curve represents the Temozolomide + Mebendazole arm and the green curve represents the CCNU+ Mebendazole arm. The numbers at risk and the participants censored (in brackets) are shown at the bottom of the graph.

The median follow up was 17.4 months (95% CI 12.7 - 22.2). On the intention to treat analysis, 68 of 88 patients had died at the time of data censoring. In the TMZ-MBZ arm 33 of 44 patients had died, yielding a median overall survival of 6.70 months (95% CI 5.50 - 9.17) and 9-month overall survival of 36.6% (95% CI 22.3 - 51.0). In the CCNU-MBZ arm, 35 of 44 patients had died, yielding a median overall survival of 6.53 months (95% CI 4.83 - 10.80) and 9-month overall survival of 45% (95% CI 29.6 – 59.2) (Figure 2). There was no difference in overall survival between the 2 arms (Hazard ratio-0.98 (95% CI 0.60–1.58); *P* = 0.90).

Three patients were deemed not to have met adequate protocol treatment requirements, hence we performed a separate per-protocol analysis on 85 patients. At the time of data censoring, 66 patients had died, 32 of 42 on the TMZ-MBZ arm and 34 of 43 on the CCNU-MBZ arm. This resulted in statistically non-significantly different median overall survival of 6.70 (95% CI 5.70- 9.17) and 6.40 (95% CI 4.70 - 10.80) [Hazard ratio 0.99 (95% CI 0.61–1.61); *P*>0.99] months for the TMZ-MBZ and the CCNU-MBZ arms. Similarly, the 9-month overall survival was not different at 35.8% (95% CI 21.3–50.6) and 46% (95% CI 30.4–60.4), for the two arms, respectively (Supplementary appendix Figure 1).

### Secondary outcomes

#### Progression-free survival

**Figure 3:**
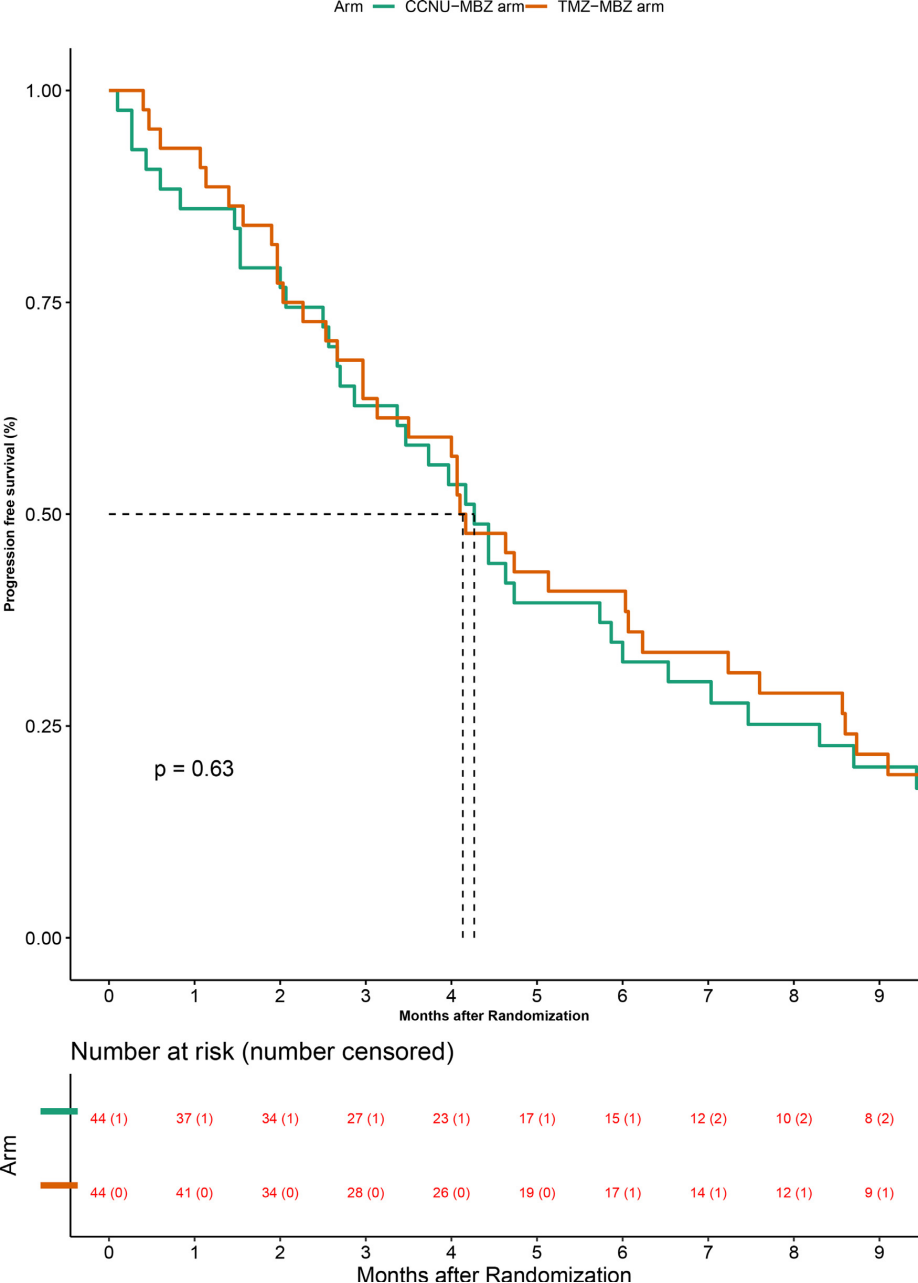
Progression-free survival-Intention to treat population. The orange curve represents the Temozolomide + Mebendazole arm and the green curve represents the CCNU+ Mebendazole arm. The numbers at risk and the participants censored (in brackets) are shown at the bottom of the graph.

On the intention to treat analysis (*n* = 88), 41 of 44 patients on the TMZ-MBZ arm, and 40 of 44 patients on the CCNU-MBZ arm had experienced an event for progression. The median progression-free survival was 4.13 months (95% CI 2.97–6.23) and 4.27 months (95% CI 2.7 - 5.87) in the TMZ-MBZ and the CCNU-MBZ arms respectively (Figure 3), which was not statistically significant (Hazard ratio 0.89 (95% CI 0.58 −1.39); *P* = 0.60).

On per-protocol analysis (*n* = 85), at the time of data censoring, 39 of 42 patients on the TMZ-MBZ arm and 39 of 43 on the CCNU-MBZ arm had an event for progression analysis. The median progression-free survival on the TMZ-MBZ and CCNU-MBZ arms was 4.13 months (95% CI 2.97–6.07), and 4.30 months (95% CI 2.70 - 5.87) respectively, which was not statistically significant (Hazard ratio 0.89 (95% CI 0.57–1.39); *P* = 0.60) (Supplementary appendix Figure 2).

#### Adverse events

The adverse events for each arm are presented in Table 2. Any grade adverse events were seen in 37 (86%; *n* = 43) and 40 (95.2%; *n* = 42) patients in TMZ-MBZ and CCNU-MBZ arms respectively. Any grade 3–5 adverse events were seen in 8 (18.6%; *n* = 43) and 4 (9.5%; *n* = 42) patients in TMZ-MBZ and CCNU-MBZ arms respectively. There was no treatment-related death in both arms.

**Table 2:** Adverse events in both arms as per CTCAE version 4.03 in accordance with per-protocol analysis. **SGOT- Serum glutamic oxaloacetic transaminase. SGPT- Serum glutamic pyruvic transaminase. TMZ -Temozolomide, MBZ-Mebendazole, CCNU-Lomustine.**

Adverse Events	TMZ-MBZ arm (*n* = 43)		CCNU-MBZ arm(*n* = 42)		P-value	
	Any grade Number (%)	Grade 3–5 Number (%)	Any grade Number (%)	Grade 3–5 Number (%)	Any grade	Grade 3–5
Anemia	13 (30.2)	1 (2.3)	17 (40.5)	1 (2.4)	0.37	1
Neutropenia	4 (9.3)	2 (4.7)	9 (21.4)	1 (2.4)	0.14	1
Thrombocytopenia	8 (18.6)	2 (4.7)	12 (28.6)	3 (7.1)	0.32	0.68
Hyponatremia	13 (30.2)	1 (2.3)	8 (19)	2 (4.8)	0.32	0.62
Hypokalemia	7 (16.3)	–	2 (4.8)	–	0.16	–
Hypomagnesemia	5 (11.6)	–	3 (7.1)	–	0.71	–
Raised SGOT	5 (11.6)	1 (2.3)	5 (11.9)	–	1	1
Raised SGPT	5 (11.6)	2 (4.7)	9 (21.4)	–	0.26	0.49
Raised Creatinine	–	–	2 (4.8)	–	0.24	–
Fatigue	26 (60.5)	1 (2.3)	33 (78.6)	–	0.099	1
Nausea	22 (51.2)	1 (2.3)	14 (33.3)	–	0.13	–
Vomiting	19 (44.2)	–	10 (23.8)	–	0.067	–
Diarrhea	1 (2.3)	–	3 (7.1)	–	0.36	–
Mucositis	3 (7.0)	–	3 (7.1)	–	1	–

#### Quality of life (QoL)

QoL data has been collected, it will be analysed and published later.

#### Compliance

Forty-three patients of 44 patients (97.7%) randomized to the TMZ-MBZ arm initiated protocol therapy. One patient refused TMZ-MBZ and took CCNU-MBZ. At the time of data censoring, 3 patients were continuing TMZ-MBZ. The reasons for the discontinuation of TMZ-MBZ in the other 41 (93.2%) are shown in Figure 1. The predominant reason for discontinuation was disease progression, seen in 33 patients (*n* = 41, 80.5%). The patient who was started on CCNU also experienced progression and is included in the 33 “progressors”

Temozolomide and mebendazole were discontinued due to adverse events in 1 patient (prolonged serum aspartate transaminase, serum alanine transaminase, and serum bilirubin abnormalities). The median number of cycles of temozolomide and mebendazole received was 4 (Interquartile range 2 - 7.75). No MBZ dose reduction was required. Temozolomide dose reduction was required in one patient after cycle 3 when the dose was decreased by 25% to 150 mg/m^2^. Three patients completed 12 cycles of TMZ-MBZ.

Forty-two of 44 patients (95.5%) randomized to CCNU-MBZ initiated protocol therapy. One patient died due to aspiration pneumonia and the other patient was lost to follow up, before the initiation of protocol therapy. At the time of data censoring, 1 patient was continuing CCNU-MBZ. The reasons for the discontinuation of CCNU with mebendazole in the other 41 (93.2%) are shown in Figure 1. The predominant reason for discontinuation was disease progression, observed in 30 patients (*n* = 41, 73.2%). CCNU and mebendazole were discontinued due to adverse events in one patient (prolonged thrombocytopenia). The median number of cycles of CCNU and mebendazole received was 3 (Interquartile range 1 - 4.75). No MBZ dose reduction was required. CCNU dose reduction was required in 5 patients. In 4 patients a dose reduction of 25% was required, while in one patient a dose reduction of 50% was required. Four patients completed 6 cycles of CCNU-MBZ.

### Impact of performance status on overall survival (Post-Hoc analysis)

Twenty-three patients enrolled had an ECOG PS of 2–3 with inferior outcomes (median OS-5.67 months (95% CI 3.33 - 6.70); HR-2.09, (95% CI 1.18 −3.73)) (Supplementary appendix Table 1). The median OS in patients with ECOG PS 0–1 was 8.87 months (95% CI 6.20 −10.80). On post-hoc analysis, 24 of the 33 patients with an ECOG PS 0–1 on the TMZ-MBZ arm had died, the median OS was 7.10 months (95% CI 4.27–10.70), and the 9-month OS was 39.6% (95% CI 22.4–56.3). In the CCNU-MBZ arm, 25 of 32 patients with an ECOG PS 0–1 had died, the median OS was 10.00 months (95% CI 4.93–12.90) and 9-month OS was 57.9% (95% CI 38.7–73.0).

### Post-progression treatment

Sixty-six of 81 (81.2%) patients experiencing a progression event received best supportive care at progression. The details of post-progression therapy are available in the supplementary appendix Table 2.

## Discussion

To the best of our knowledge this is the first randomized trial addressing the role of mebendazole in recurrent glioblastoma. Unfortunately, the combination of CCNU-MBZ and TMZ-MBZ failed to improve upon the 9-month OS benchmark of 55%. The pivotal EORTC 26101 study had a median PFS of 1.5 months in the CCNU arm, and 4.2 months in the CCNU and bevacizumab arm. In our trial, both of the MBZ arms showed promising median PFS of 4.13 and 4.27 months respectively. However, our patient population was significantly younger and with a much higher rate of IDH mutations.

The low overall survival seen in our study could be attributed to multiple factors. First, the study was conducted in a real-world setting where nearly one-third of patients have poor PS at the time of disease progression.^16^ We allowed the inclusion of ECOG PS 2–3 patients who constituted 28.6% (23 out of 88) of the cohort, this was significantly higher than the EORTC 26101 where only 10.2% of patients had ECOG PS 2 and there were no patients with PS 3.^8^ Similarly, in the BELOB study,^9^ only 11.5% of patients had ECOG PS 2 and PS 3 patients were excluded. The 9-month OS in patients with ECOG PS 0–1 in our study was 39.6% and 57.9% in TMZ-MBZ and CCNU-MBZ arms, respectively. This suggests that if we had restricted our eligibility and maintained the protocol P0 and P1 rules, CCNU-MBZ could have breached the 55% threshold. Although promising, we need to interpret the results of the subgroup analysis of patients with an ECOG PS of 0–1 with the caveat that this was a post-hoc analysis. It included 33 patients in the TMZ-MBZ group and 32 in the CCNU-MBZ group, which is less than the pre-specified sample size of 44 patients for each arm. Mebendazole has shown promising activity in combination with TMZ in newly diagnosed high-grade gliomas with a median OS of 21 months (95% CI: 14.3–31.2), with 41.7% of patients alive at 2 years and 25% at 3 and 4 years.^12^ In patients who received more than a month of MBZ, the median PFS was 13.1 months (95% CI: 8.8–14.6 months). Thus, in well-selected fit patients, the addition of MBZ to chemotherapy has the potential to improve survival, even in the recurrent setting.

A second explanation for the poor overall survival is limited access to other salvage therapy such as bevacizumab, TTF therapy, and newer investigational agents in low and middle-income countries (LMICs).^16^ This is reflected in our study, where 81.2% of patients received best supportive care at progression. This is in sharp contrast with the EORTC 26101 trial where 65.9% of patients on the CCNU arm and 53% on the CCNU plus Bevacizumab arm received post-progression therapy.^8^ The corresponding figures in the BELOB study varied from 38 to 57%.^9^ This lack of post-progression therapy could have contributed to the lower OS observed in our study.

Thirdly, the inclusion criteria in our study specified that the patients should be unsuitable for re-radiation. This is a cohort of patients who have poor outcomes. In the BELOB study, re-radiation post-progression was performed in 20 to 24% of patients on various arms.^9^ The corresponding figures in the EORTC study were 9.6 to 13.8% across the 2 arms.^8^ The inclusion of patients with poor performance status and unsuitable for re-radiation seems to have impacted the OS.

Another reason for failing to attain the OS objective could be over-ambitious goal-setting. The benchmark of 9-month OS of 35% as P0 and 55% as P1 was set based on the BELOB study. However, as highlighted above our study was conducted in a very different patient population, reflecting the less well-selected real-world patients, including those with poor PS, not eligible for re-irradiation, and those without access to further salvage options, etc. After we had launched our trial, Menon et al., published a real-world analysis demonstrating 25% 9-month OS in a recurrent GBM population not eligible for reirradiation. In our trial, we had assumed a P0 of 35%, and the P1 was set with an absolute delta of 20%; with the new data from Menon et al., we would in hindsight, have set the P0 at 25% and seeking a 20% absolute delta, the P1 at 45%.^5^

The study was not designed to compare whether CCNU or TMZ in combination with MBZ was superior to the other, but to determine whether the addition of MBZ to TMZ or CCNU improved outcomes in comparison with historical data. The two arms were treated as separate patient cohorts, but patients were randomly assigned so that recruitment was even and baseline characteristics were balanced between the two cohorts.

This study has its limitations. This was a single-center study conducted at a premier cancer center in India and catering to patients across the nation and often draws those with no other options. The lack of a CCNU alone and a TMZ alone arm is a potential drawback of this study. The presence of these arms would have allowed us to make firmer conclusions regarding the role of mebendazole even with the recruitment of poor PS patients and limited access to post-progression therapy. At the time of planning the study, we had a discussion in our neuro-oncology disease management group (DMG) on whether to include a CCNU/TMZ alone arm in the study. Considering the dismal outcomes with CCNU and TMZ alone, the neuro-oncology DMG decided that it would be better to offer the benefit of the addition of Mebendazole to CNNU/TMZ if any, to all patients and to use historical results as control.

The strength of the study is that it was conducted in a real-world setting in a LMIC and it attempted to repurpose a drug that is administered orally, available universally, inexpensive with multiple generics and does not require cold storage. We therefore speculatively hypothesize that the CCNU-MBZ might have the modest potential for improving outcomes in ECOG PS 0–1 recurrent GBM and warrants further exploration.

In conclusion, the addition of mebendazole to temozolomide or CCNU failed to achieve the set benchmark of 9-month OS of 55% in recurrent GBM. This was possibly due to 28.6% of patients having poor PS of 2–3, as well other limitations. The post-hoc analysis revealed that patients with ECOG PS 0–1 in the CCNU-MBZ arm had a 9-month OS of 57.9% and this needs further evaluation.

## Contributors

Conceptualisation-VP, RJ

Funding acquisition- VP

Data Acquisition: All authors

Investigation: All authors

Project Administration: VP, NM, MJ, SP, ZP

Verified the data: VP, NM, SP, ZP

Formal Analysis: VP

Wrote the first draft of the manuscript: VP, NM

Writing- Review and editing: All authors.

All authors had full access to all the data in the study and shared final responsibility for the decision to submit for publication.

## Funding

Brain Tumor Foundation (BTF) of India, Indian Cooperative Oncology Network (ICON), and India Cancer Research Consortium (ICRC) under ICMR (Indian Council of Medical Research).

### Data sharing statement

De-identified participant data will be made available with the publication of this manuscript by the corresponding author (VP) for any scientific purpose with a signed data access agreement.

## Declaration of interests

VP reports research grants from Brain Tumor Foundation (BTF) of India, grants from Indian Cooperative Oncology Network (ICON), grants from India Cancer Research Consortium (ICRC) under ICMR (Indian Council of Medical Research), during the conduct of the study; and research grants from Janssen & Janssen, Astra Zeneca, Intas Pharmaceuticals, Natco Pharma, Cadila Healthcare, Eisai, and Novartis outside the submitted work. NM reports a research grant from Astra Zeneca outside the submitted work. All grants were paid to the institution. All other authors report no conflicts of interest.

## Appendix Supplementary materials

Image, application 1

Image, application 2
