# Supplementary material for: Mebendazole plus lomustine or temozolomide in patients with recurrent glioblastoma: A randomised open-label phase II trial
Source: eClinicalMedicine. 2022 May 27;49:101449. doi: 10.1016/j.eclinm.2022.101449 (PMC9156991; doi:10.1016/j.eclinm.2022.101449)
Supplement: Supplementary file 1 [file mmc1.docx]

**Caption for Supplementary Material**

1. Supplementary Appendix
2. Study Protocol
