# Supplementary material for: Mebendazole plus lomustine or temozolomide in patients with recurrent glioblastoma: A randomised open-label phase II trial
Source: eClinicalMedicine. 2022 May 27;49:101449. doi: 10.1016/j.eclinm.2022.101449 (PMC9156991; doi:10.1016/j.eclinm.2022.101449)
Supplement: Supplementary file 2 [file mmc2.pdf]

1. Supplementary Appendix

Table 1

	**Number**	**HR**	**95% CI of HR**	**P-value**
**Age (continuous variable)**	88	0.978	0.954-1.0	0.0549
**Gender**				
Male	65	0.92	0.523-1.618	0.7718
Female	23	Reference		
**ECOG PS**				
PS 0-1	65	Reference		
PS 2-3	23	2.092	1.175-3.731	0.012
**MGMT status**				
Unmethylated or Unknown	73	0.79	0.386-1.616	0.518
Methylated	15	Reference		
**IDH status**				
Mutated	32	0.591	0.343-1.017	0.057
Unknown	56	Reference		
**Arm**				

TMZ-MBZ	44	0.978	0.596-1.606	0.9311
CCNU-MBZ	44	Reference		

Supplementary appendix Table 1- Table depicting the factors impacting overall survival on intention to treat analysis. ECOG PS: - Eastern Cooperative Oncology Group performance status, IDH - Presence of Isocitrate dehydrogenase 1 and 2 mutations, MGMT- Methylation of the O (6)-Methylguanine-DNA methyltransferase. HR-Hazard ratio. TMZ- Temozolomide, MBZ-Mebendazole, CCNU-Lomustine.

Table 2

**Characteristic**	**TMZ-MBZ arm (n=44)**	**CCNU-MBZ arm(n=44)**
No progression event	3(6.8%)	4(9.1%)
Best supportive care	28(63.6%)	28(63.6%)
Bevacizumab	6(13.6%)	8(18.2%)
CCNU	6(13.6%)	-
Nivolumab	-	1(2.3%)
Bevacizumab followed by Erdafitinib	1(2.3%)	-
Osimertinib and Bevacizumab	-	1(2.3%)
Dabrafenib with Trametinib	-	1(2.3%)
Temozolomide	-	1(2.3%)

Supplementary Table 2- Table showing post-progression therapy. CCNU-Lomustine.

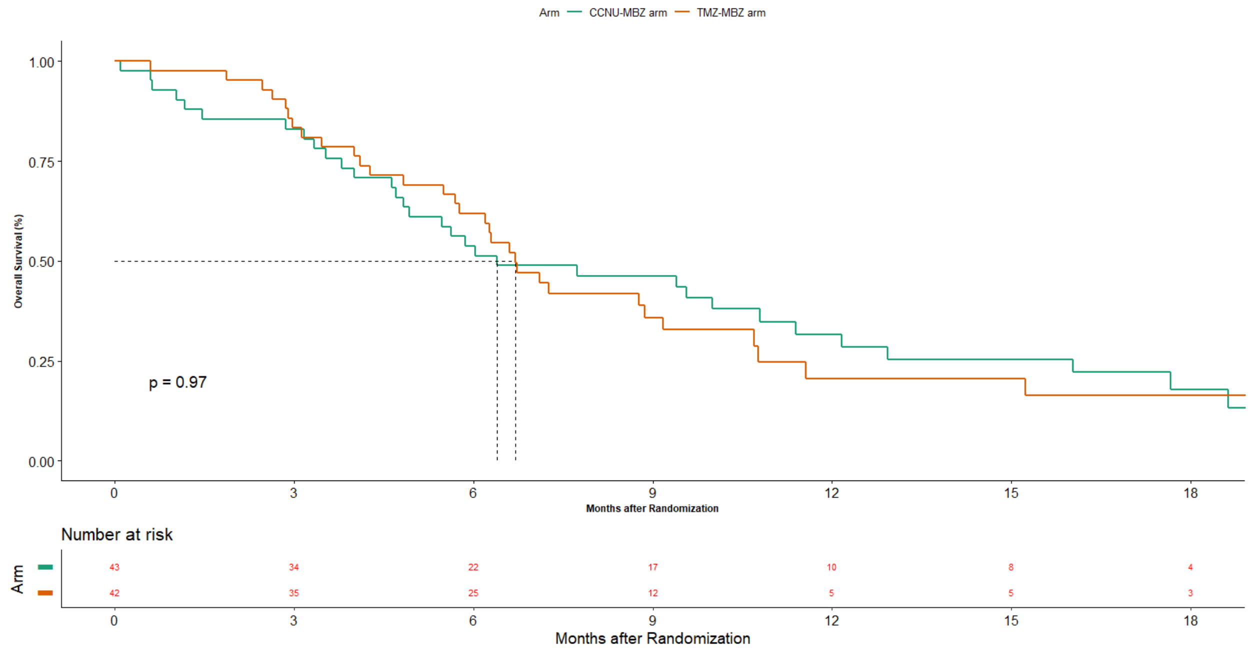

Supplementary Figure 1- Overall survival-Per-Protocol graph

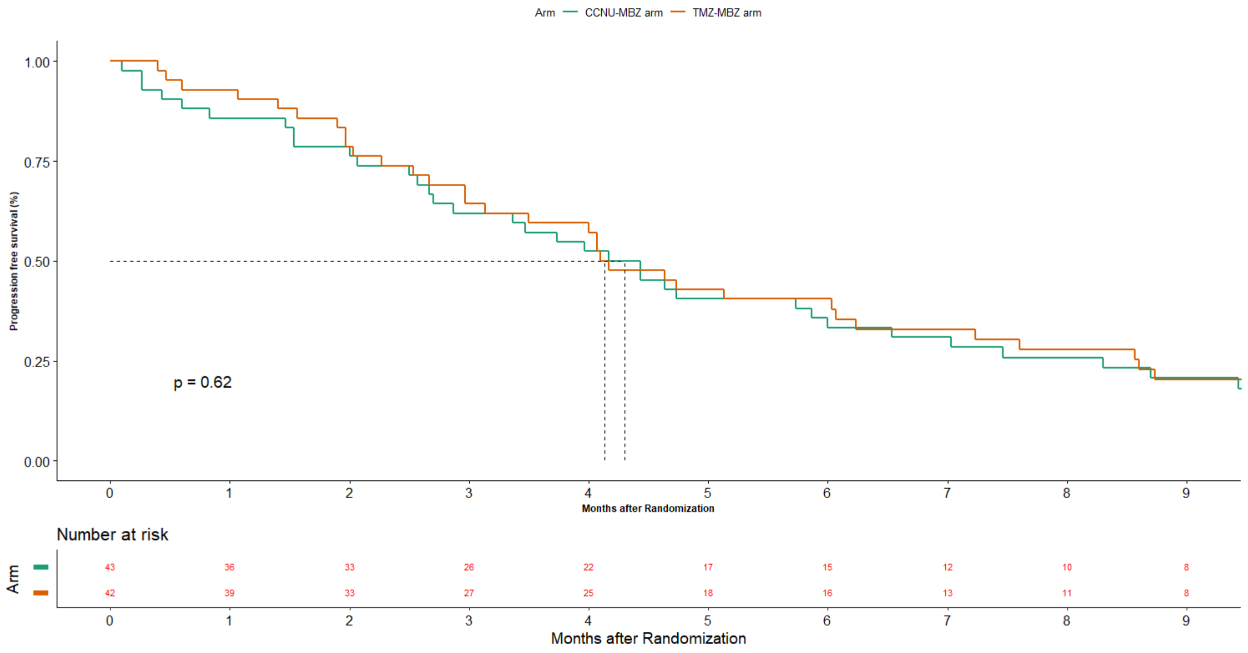

Supplementary appendix Figure 2- Progression-free survival-Per Protocol graph

2. Study Protocol

Reverse Swing-M: Repurposing of Mebendazole in Recurrent Glioblastoma.

Table of contents

1 Protocol schema 10

2 Background 11

2.1 Management of recurrent high grade glioma 11

2.1.1 Re Surgery 11

2.1.2 Re-radiation 11

2.1.3 Chemotherapy 12

2.2 Mebendazole in glioma 13

2.2.1 In vivo and animal data 13

2.3 Pharmacology of mebendazole 13

2.3.1 Structure 14

2.3.2 MTD in benign disease 14

2.3.3 Pharmacokinetics 14

2.3.3.1 Absorption 14

2.3.3.2 Distribution 14

2.3.3.3 Metabolism 14

2.3.3.4 Excretion 15

2.3.3.5 Steady-state Pharmacokinetics 15

2.3.3.6 Polymorphism 15

2.3.3.7 Side effects

2.3.4 Mebendazole in cancer 15

3 Rationale 16

4 Hypothesis 16

5 Objectives 17

5.1 Phase 1 17

5.1.1 Primary objective 17

5.2 Phase 2 17

5.2.1 Primary objectives for recurrent glioblastoma cohorts 17

5.2.2 Secondary objectives 17

6 Trial design 17

7 Study setting 18

8 Eligibility criteria 18
- 8.1 Inclusion criteria 18
- 8.2 Exclusion criteria 19

9 Intervention 19
- 9.1 Prescreening 19
- 9.2 Screening 20
- 9.3 Baseline assessment 20
- 9.4 Phase 1 20
  - 9.4.1 Summary 20
  - 9.4.2 Definitions used for outcome analysis in Phase 1 20
    - 9.4.2.1 Maximum tolerable dose (MTD) 20
    - 9.4.2.2 Dose limiting toxicity (DLT) 21
    - 9.4.2.3 Adverse events considered as DLT 21
    - 9.4.2.4 Recording of DLT 21
  - 9.4.3 Dose levels 21
  - 9.4.4 Schema of phase 1 22
  - 9.4.5 Selection of Arm 23
    - 9.4.5.1 Glioblastoma histology 23
  - 9.4.6 Administration of drug 23
  - 9.4.7 Companion drug in Arm A1, B1 & C1 23
    - 9.4.7.1 Arm A1 23
      - 9.4.7.1.1 Radiation 23
      - 9.4.7.1.2 Temozolomide 23
      - 9.4.7.1.3 Duration of treatment 24
      - 9.4.7.1.4 Compliance criteria 24
      - 9.4.7.1.5 Adverse events recording 24
    - 9.4.7.2 Arm B1 25
      - 9.4.7.2.1 CCNU 25
      - 9.4.7.2.2 Duration of treatment 25
      - 9.4.7.2.3 Compliance criteria 25
      - 9.4.7.2.4 Adverse events recording 25
    - 9.4.7.3 Arm C1 26
      - 9.4.7.3.1 Temozolomide 26
      - 9.4.7.3.2 Duration of treatment 27
      - 9.4.7.3.3 Compliance criteria 27
      - 9.4.7.3.4 Adverse events recording 27
  - 9.4.8 Criteria for starting first cycle 27
  - 9.4.9 Dose modifications 28

9.4.9.1 Mebendazole 28
9.4.9.2 Temozolomide 28
9.4.9.3 CCNU 28
9.4.9.3.1 Dose modification for hepatic dysfunction 28
9.4.9.3.2 Dose modification for renal dysfunction 28
9.4.9.3.3 Dose modification for myelosuppression 29
9.4.10 Accompanying medications 29
9.4.11 Criteria for discontinuing protocol 29
9.4.12 Post protocol care 29
9.5 Phase 2 30
9.5.1 Summary of phase 2 30
9.5.2 Selection of Arm 30
9.5.2.1 Glioblastoma histology 30
9.5.3 Administration of drug 30
9.5.3.1 Mebendazole 30
9.5.4 Companion drug in Arm A2, B2 ,C2 31
9.5.4.1 Arm A2 31
9.5.4.1.1 Radiation 31
9.5.4.1.2 Temozolomide 31
9.5.4.1.3 Duration of treatment 31
9.5.4.1.4 Compliance criteria 31
9.5.4.1.5 Adverse events recording 31
9.5.4.2 Arm B2 32
9.5.4.2.1 CCNU 32
9.5.4.2.2 Duration of treatment 32
9.5.4.2.3 Compliance criteria 33
9.5.4.2.4 Adverse events recording 33
9.5.4.3 Arm C2 33
9.5.4.3.1 Temozolomide 33
9.5.4.3.2 Duration of treatment 34
9.5.4.3.3 Compliance criteria 34
9.5.4.3.4 Adverse events recording 34
9.5.5 Criteria for starting first cycle 34
9.5.6 Criteria for starting second cycle (Arm B2,C2) or continuing TMZ in Arm A2 35
9.5.7 Dose modifications 35
9.5.8 Accompanying medications 35
9.5.9 Documentation of response and quality of life 35
9.5.9.1 Response assessment 35
9.5.9.2 Quality of life assessment 36
9.5.10 Criteria for discontinuing protocol 36
9.5.11 Post protocol care 36

10 Outcomes 36
10.1 Phase 1 objective 36
10.2 Phase 2 objective 36
10.2.1 Primary outcome for recurrent glioblastoma cohorts : 9 month OS 36
10.2.2 Secondary outcomes 37
11 Participant timeline 37
11.1 Phase 1 37
11.2 Phase 2 38
12 Sample size 39
13 Data collection method and management 39
14 Data monitoring 40
15 Harms 40
16 Auditing 40
17 Ethics approval 40
18 Protocol amendments 40
19 Consent or assent 41
20 Confidentiality 41
21 Declaration of interests 41
22 Access to data 41
23 Dissemination policy 41
24 References 42

1 Protocol schema

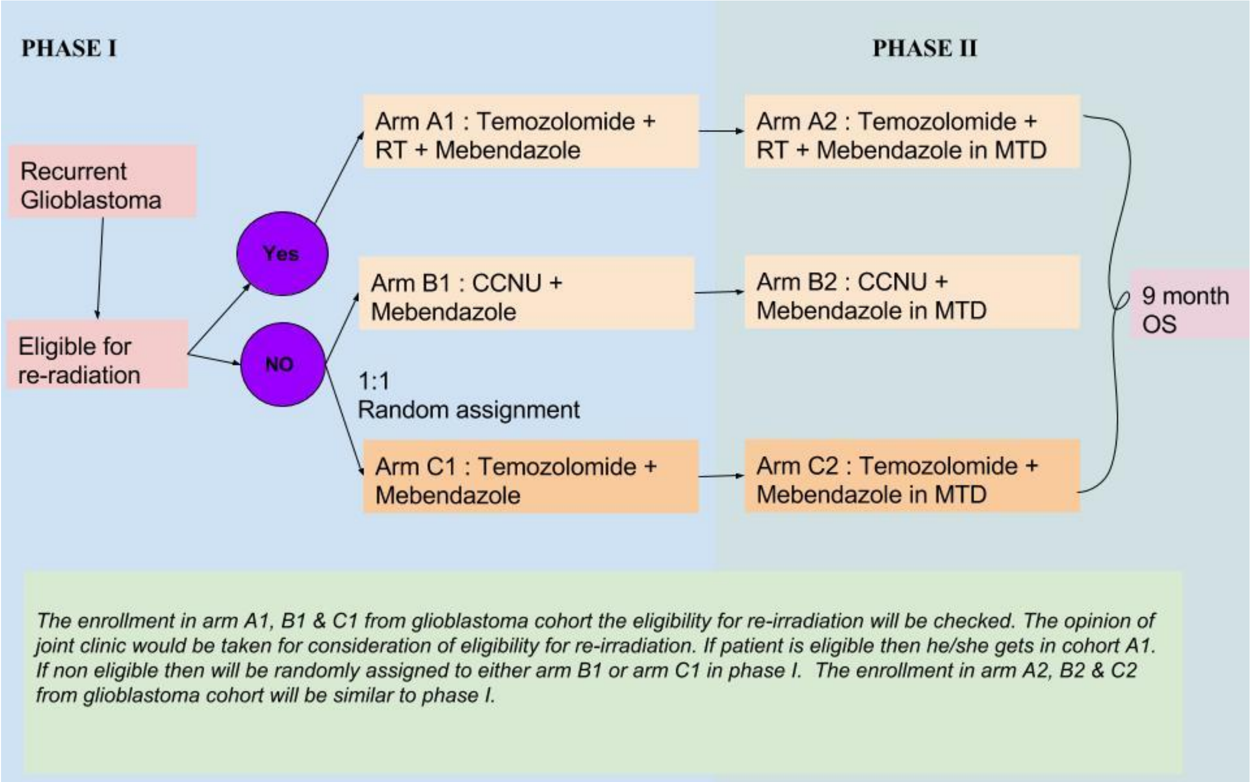

Figure 1 : Protocol schema

2 Background

2.1 Management of recurrent high-grade glioma

Recurrence in high grade glioma is inevitable. For patients with glioblastoma (GBM) treated with the current standard of care (maximal safe resection, fractionated external beam radiotherapy, and concurrent and adjuvant temozolomide) in the European Organisation for Research and Treatment of Cancer–National Cancer Institute of Canada randomized trial, 2- and 5-year progression-free survivals (PFSs) of only 11% and 4%, respectively, were observed with less than 10% of patients surviving more than 5 years from diagnosis [1,2]. Similarly, in NOA-08 study, the median overall survival was 8.6 months (95% CI 7·3–10·2) in the temozolomide group versus 9·6 months (8·2–10·8) in the radiotherapy group (hazard ratio [HR] 1.09, 95% CI 0·84–1·42, p non-inferiority=0·033). Median event-free survival (EFS) was similar between the temozolomide and radiotherapy groups (3·3 months [95% CI 3·2–4·1] vs 4·7 [4·2–5·2]; HR 1.15, 95% CI 0·92–1·43, non-inferiority=0·043)[3].

The gold standard for diagnosis of recurrent disease is, a definitive histologic confirmation. However, before performing a biopsy to establish or deny gross recurrence, it is essential to ask whether the value of making the diagnosis outweighs the risk of the procedure. Practically the diagnosis of such recurrence is generally based on clinical suspicion and radiological confirmation[4].

2.1.1 Re Surgery

Management of recurrent high grade requires a multidisciplinary approach. However surgical resection being potentially diagnostic and therapeutic. In particular, surgery tends to be most beneficial when there is a well demarcated lesion involving non eloquent brain, producing a symptomatic mass effect on normal brain structures[4]. However, reoperation may be complicated by several factors. First, the site of recurrence is at or near the resection bed, and this volume has typically received a full dose of radiation during the initial course of treatment, potentially impairing wound healing. Second, the goal of the initial glioma surgery is to achieve maximal safe resection and, consequently, surgical margins may often abut eloquent areas. Thus, for recurrences near the resection cavity, the extent of reoperation may be severely constrained. Practically the re surgery as an option is limited to a minority of patients who have higher Karnofsky performance status, lower age, and smaller, more readily resectable recurrent tumors. However the median OS still ranges between 5.3 -8.3 months in various series[4].

2.1.2 Re-radiation

All high-grade gliomas would have received partial brain radiation to a dose of 60Gy in total in 1.8-2.0 Gy per fractions. Hence in such setting of previous partial-brain irradiation and recurrence within the volume of the brain receiving an initial high dose of radiation (the most common scenario), it is difficult to administer another “conventional” course of irradiation to the recurrent lesion and margin without risking adverse toxicity. Thus, hypofractionated stereotactic

radiotherapy (HFSRT) or stereotactic radiosurgery (SRS) or conventional is employed in case of a limited volume recurrence occurring after 11/2- 2 years of previous radiation. However, such treatments also present logical inconsistencies. First, as most patients with recurrent disease initially received a high-dose radiation to the area of recurrence, it is not clear why a second treatment should be any more effective than the first. At the very least, one would expect a “narrower” therapeutic window due to the effects of the initial irradiation. Second, an SRS boost as part of the initial treatment of malignant gliomas showed no benefit over conventional radiotherapy alone in RTOG 9305[5]. Third, as malignant gliomas have a diffuse, broadly infiltrative component in addition to discrete nodular disease, it is unclear why a strictly “local” treatment of the nodules alone should substantially alter the outcome. Despite these apparent limitations, SRS, SRT, and even conventionally fractionated RT appear to provide reasonable OS in comparison with that by chemotherapy alone for the treatment of recurrent glioma, with median OS ranging from 5-13 months.[4] However as the fact that focal radiotherapy requires a discrete target exclude those patients with diffuse brain disease and those who recur within 1-1 ½ year post initial RT. Unfortunately, 60-80% of patients would have either of the above criteria making them ineligible for re-radiation.

2.1.3 Chemotherapy

Salvage chemotherapy is the commonest option utilized in this setting. Multiple agents have been used however consistent improvements in survival have not been proven. Table 1 gives a list of results of CCNU, temozolomide and bevacizumab. In addition to the molecules shown in table 1, lapatinib, sirolimus, temsirolimus, pazopanib, Nintedanib, glufosfamide, imatinib, erlotinib, IFNβ, IFNβ with cis-retinoic acid, menogaril, difluoromethylornithine and sagopilone have been used with dismal results.[4] At present bevacizumab is the US FDA recommended treatment. This recommendation is based on multiple phase 2 single arm studies.[6,7] In developing countries either due to cost or its contraindications in multiple patients its administration is infeasible. Further in the recently conducted BELOB study the bevacizumab arm either alone or in combination with CCNU was not statistically superior to CCNU alone. It's unclear at present whether bevacizumab alone or in combination is better than CCNU or temozolomide[7]. Hence in clinical practice both CCNU and rechallenge with temozolomide is used in addition to bevacizumab. ***In view of the unsatisfactory results with these agents where the median OS is between 6.0-8.0 months there is an urgent felt need for newer agents.***

Authors	Arm	Median OS in each arm in months
Wick et al[8]	Enzastaurin<br>CCNU	6.6 months<br>7.1 months
Batchelor et al[9]	Cediranib<br>Cediranib +CCNU<br>CCNU +Placebo	8.0 months<br>9.4 months<br>9.8 months
Taal et al[7]	Bevacizumab	8.0 months

	CCNU<br>Bevacizumab +CCNU	8.0 months<br>16.0 months
Brandes et al[10]	Galunisertib<br>Galunisertib +CCNU<br>CCNU +Placebo	8.0 months<br>6.7 months<br>7.5 months
Moller et al[6]	Bevacizumab + Irinotecan	7.9 months
Franceschi E et al[11]	CCNU + dasatinib	6.4 months
Galanis E et al[12]	Bevacizumab + sorafenib	5.6 months
Kreisl TN et al[13]	Sunitinib	9.4 months
Kreisl TN et al[14]	Vandetanib	6.3 months
Balmaceda C et al[15]	Temozolomide	8.8 months
Omuro A et al [16]	Temozolomide	7.0 months

Table 1 : Details of multiple chemotherapy studies in second line setting in gliomas

2.2 Mebendazole in glioma

2.2.1 In vivo and animal data

Bai et al serendipitously observed that fenbendazole, a benzimidazole anthelmintic used to treat pinworm infection, inhibited brain tumor engraftment. Subsequent they in their in vitro and in vivo experiments with benzimidazoles identified mebendazole as the more promising drug for GBM therapy.[17]

In GBM cell lines, mebendazole has displayed cytotoxicity, with half-maximal inhibitory concentrations ranging from 0.1 to 0.3 µM. Mebendazole disrupts microtubule formation in GBM cells, and in vitro activity correlates with reduced tubulin polymerization. In addition, Bai et al have also shown that mebendazole significantly extends mean survival up to 63% in syngeneic and xenograft orthotopic mouse glioma models.[18]

2.3 Pharmacology of mebendazole

Mebendazole is an anthelmintic drug. It has a broad spectrum of anthelmintic activity and is effective in the treatment of single or mixed helminthic infestations. Clinical studies have shown it to be effective in the treatment of Enterobius vermicularis (pinworm); Ascaris lumbricoides (roundworm); Trichuris trichiura (whipworm); Ancylostoma duodenale and Necator americanus (hookworm). It has also been used to treat infestations due to Strongyloides stercoralis (threadworm) and Taenia solium (large tapeworms)[19].

2.3.1 Structure

Chemical name: Methyl 5-benzoyl-2-benzimidazole carbamate

Formula: $C_{16}H_{13}N_3O_3$

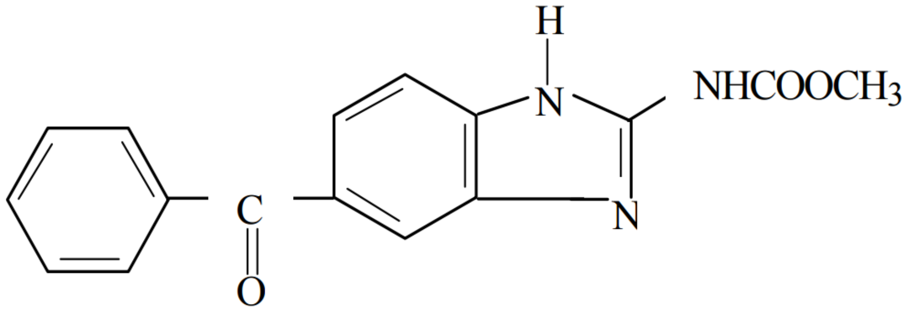

Figure 2: Chemical structure of mebendazole

2.3.2 MTD in benign disease

Dose in pinworm: 100 mg single tablet
Dose in round or hookworm: 100 mg BD for 3 days
Dose in cystic echinococcosis: 40-50 mg/kg/ day for at least 3-6 months
Dose in alveolar echinococcosis: 40-50 mg/kg/ day for at least 24 months

2.3.3 Pharmacokinetics

2.3.3.1 Absorption

Following oral administration, < 10% of the dose reaches the systemic circulation, due to incomplete absorption and extensive pre-systemic metabolism (first-pass effect). Maximum plasma concentrations are generally seen 2 to 4 hours after administration. Dosing with a high-fat meal leads to a modest increase in the bioavailability of mebendazole.

2.3.3.2 Distribution

The plasma protein binding of mebendazole is 90 to 95%. The volume of distribution is 1 to 2 L/kg, indicating that mebendazole penetrates areas outside the vascular space. This is supported by data in patients on chronic mebendazole therapy (e.g., 40 mg/kg/day for 3–21 months) that show drug levels in tissue.

2.3.3.3 Metabolism

Orally administered mebendazole is extensively metabolized primarily by the liver. Plasma concentrations of its major metabolites (amino and hydroxylated amino forms of mebendazole) are substantially higher than those of mebendazole. Impaired hepatic function, impaired

metabolism, or impaired biliary elimination may lead to higher plasma levels of mebendazole. Concomitant treatment with cimetidine inhibits is metabolism in the liver, resulting in increased plasma concentrations of the drug, especially during prolonged treatment.

2.3.3.4 Excretion

Mebendazole, the conjugated forms of mebendazole, and its metabolites likely undergo some degree of enterohepatic recirculation and are excreted in the urine and bile. The apparent elimination half-life after an oral dose, ranges from 3 to 6 hours in most patients.

2.3.3.5 Steady-state Pharmacokinetics

During chronic dosing (e.g., 40 mg/kg/day for 3–21 months), plasma concentrations of mebendazole and its major metabolites increase, resulting in approximately three-fold higher exposure to steady-state compared to single dosing.

2.3.3.6 Polymorphism

Mebendazole shows preclinical efficacy in models of glioblastoma. Three different mebendazole polymorphs (A, B, and C) exist. The polymorph B and C both increase survival in a GL261 glioma model, but B exhibited greater toxicity. Polymorph A shows no benefit. Polymorph B and C both reach concentrations in the brain that exceeded the IC50 in GL261 cells 29-fold. In addition, polymorph C demonstrated an AUC0–24h brain-to plasma (B/P) ratio of 0.82, whereas B showed higher plasma AUC and lower B/P ratio. In contrast, polymorph A presented markedly lower levels in the plasma and brain. ***So among mebendazole polymorphs, C reaches therapeutically effective concentrations in the brain tissue and tumor with fewer side effects, and is the better choice for brain cancer therapy.***[20]

2.3.3.7 Side effects

At the recommended dose for anthelmintic treatment (100 mg BD for 3 days), mebendazole is generally well tolerated. However, patients with high parasitic burdens have manifested diarrhea, vomiting, and/or abdominal pain. Other adverse reactions reported were drowsiness, itching, headache, flatulence, dizziness, increased SGOT, SGPT, alkaline phosphatase, and BUN. Eosinophilia and decreased hemoglobin and/or white cell count, hematuria, and cylindruria have been reported. However, most of these adverse events are related to parasitic infection.[19]

Long term use of mebendazole (average 24 months) is also well tolerated. This data is from an open-labelled observational study in which alveolar echinococcosis was treated with mebendazole. Three patients (of 17), the treatment was changed to albendazole due to intolerable side effects (reversible alopecia, psychological disturbance, and drop in performance.[21]

2.3.4 Mebendazole in cancer

In an abstract presented at SNO conference (abstract number NO-16), Okamura et have presented results of long-term use of mebendazole. In their study thirteen patients with high-grade gliomas had previously failed standard therapy (surgery followed by adjuvant radiotherapy and temozolomide) and had clear evidence of tumor progression were selected. All patients received mebendazole with oral dose of 800 mg/day The primary end point of the study was to determine the safety. Median age was 62 years, and 11 patients with glioblastoma, and 3 with anaplastic astrocytoma. Dose escalations to maximum of 4200 mg/day were applied for 3 GBM patients. No dose-limiting non-CNS toxicity were encountered. The acute adverse reactions were grades I -II nausea, vomiting, diarrhea, and liver dysfunction, but they were all relieved after symptomatic treatment. The toxicity of more than grade III was not observed. Of the 13 patients, 10 had received continuously with monthly temozolomide treatments, but 3 had not received any treatments other than mebendazole. Median mebendazole treatment period was 18 weeks. Four had complete response, 3 had stable disease, and 6 had progressive disease. The median time to progression was 16 weeks. The preliminary results demonstrated that mebendazole for recurrent high-grade glioma appears to be safe and effective.

3 Rationale

In view of dismal results of currently available treatment options in recurrent high-grade gliomas, it is necessary to actively research innovative options in this situation. Mebendazole has provided promising results in in vivo and animal models. In addition, over a wide dose range extending up to 50 mg/kg/day for 2 years (3000 mg per day, assuming a weight of 60 kg) mebendazole safety is already proven. Hence it is safe and necessary to study its efficacy in humans. At present the maximum tolerable dose of mebendazole in humans along in combination with different drugs is unknown. Hence this study is planned for determining the MTD of mebendazole and its efficacy along different drugs in recurrent glioblastoma.

4 Hypothesis

Null hypothesis for phase 1: The maximum tolerable dose of mebendazole is not more than 1500 mg / day in combination with various drugs in recurrent glioblastoma
Null hypothesis for phase 2 in glioblastoma: The combination of mebendazole with various drugs or radiation would not improve the 9-month OS beyond 55%.

5 Objectives

5.1 Phase 1

5.1.1 Primary objective

To determine the maximum tolerable dose of mebendazole in below mention combinations

1. Mebendazole with temozolomide (200 mg /m^2, day 1-day 5, 28-day cycle)
2. Mebendazole with CCNU (110 mg/m^2, day 1, 42-day cycle)
3. Mebendazole with temozolomide (75mg/m^2 daily as radiosensitizer) with curative re-radiation.

5.2 Phase 2

5.2.1 Primary objectives for recurrent glioblastoma cohorts

To estimate the 9-month overall survival in following cohorts of recurrent glioblastoma

1. Mebendazole in MTD with temozolomide (200 mg /m^2, day 1-day 5, 28-day cycle)
2. Mebendazole in MTD with CCNU (110 mg/m2 , day 1 , 42 day cycle)
3. Mebendazole in MTD with temozolomide (75mg/m2 daily as radiosensitizer) with curative re-radiation.

5.2.2 Secondary objectives

1. To estimate the median PFS in each cohort
   a. Mebendazole in MTD with temozolomide (200 mg /m^2, day 1-day 5, 28 day cycle)
   b. Mebendazole in MTD with CCNU (110 mg/m^2, day 1, 42 day cycle)
   c. Mebendazole in MTD with temozolomide (75mg/m2 daily as radiosensitizer) with curative re-radiation.
2. To estimate the improvement in quality of life in all cohorts at baseline, at 3 months and 6 months
3. To estimate the cumulative incidence of grade 3-4 adverse events during treatment in accordance with CTCAE version 4.03

6 Trial design

Multi-arm, Multistage, randomized , open label, explanatory, phase 1 going to phase 2 study.

7 Study setting

The study would be conducted in the department of medical oncology, in the neuro DMG outpatient wing.

8 Eligibility criteria

8.1 Inclusion criteria

1. Participants must have recurrent glioblastoma (for randomization in either Arm A1, B1,C1 or in Arm A2, B2, C2)
    a. The diagnosis of recurrent glioblastoma could be based on any of the below mentioned features
        i. Histopathological confirmation of recurrence: Defined as histopathology or FNA showing a viable glioblastoma
        ii. Clinico-radiological features: Unequivocal clinical or radiological features suggesting recurrence or progression
2. Age: Any age above 18 years. No maximum age.
3. ECOG performance status ≤3
4. Participants must have normal organ and marrow function as defined below:
    a. Leukocytes ≥2,000/mcL or ANC ≥ 1500/mcL
    b. Platelets ≥100,000/mcL
    c. Total bilirubin < 1.5 × institutional upper limit of normal
    d. AST(SGOT)/ALT(SGPT) ≤2.5 × institutional upper limit of normal
    e. Calculated Creatinine clearance > 30 ml/min
5. The time since last chemotherapeutic investigational treatment should be more than 3 weeks
6. The effects of mebendazole on the developing human fetus are teratogenic. Hence women of childbearing potential and men must agree to use adequate contraception (hormonal or barrier method of birth control; abstinence) prior to study entry and for the duration of study participation. Should a woman become pregnant or suspect she is pregnant while she or her partner is participating in this study, she should inform her treating physician immediately. Men treated or enrolled on this protocol must also agree to use adequate contraception prior to the study, for the duration of study participation, and 6 months after completion of protocol.
7. Both men and women of all races and ethnic groups are eligible for this trial.
8. Willing and able to comply with all study requirements, including treatment (able to swallow tablets), able to be followed up at regular intervals and/or nature of required assessments (e.g. ability to undergo video follow up)
9. Ability to understand and the willingness to sign a written informed consent document

8.2 Exclusion criteria

1. Participants who had life threatening complications either during CTRT with temozolomide or during adjuvant cycles of temozolomide
2. Participants who are receiving any other investigational agents.
3. Within 4 weeks of administration of chemotherapeutic agent
4. Failure within 3 months of stopping temozolomide
5. Patients with QTc prolongation defined as QTc interval greater than 440 ms in males and 480 ms in females in view of risk of sudden cardiac death associated with use of Ondansetron.
6. History of allergic reactions attributed to compounds of similar chemical or biologic composition to any agents used in study.
7. Patients who have taken any benzimidazole (ABZ, flubendazole, thiabendazole, fenbendazole, triclabendazole, etc.) within the last 3 months
8. Uncontrolled intercurrent illness including, but not limited to, hypertension, tuberculosis, diabetes, ongoing or active infection, symptomatic congestive heart failure, unstable angina pectoris, cardiac arrhythmia, renal failure (on dialysis), active gastrointestinal bleeding, cerebrovascular accidents, inflammatory bowel disease, known hyperkalemia (CTCAE version 4.02 grade 3 or above which is persistent over 1 week) or psychiatric illness/social situations that would limit compliance with study requirements.
9. Pregnant women and breastfeeding women are excluded from this study because temozolomide has the potential for teratogenic or abortifacient effects. Because there is an unknown but potential risk for adverse events in nursing infants. These potential risks may also apply to other agents used in this study.
10. Patients with human immunodeficiency virus (HIV), hepatitis B surface antigen or hepatitis C positive; or with a history of chronic active hepatitis or cirrhosis
11. Patients with a history of any medical or psychiatric condition or laboratory abnormality that in the opinion of the investigator may increase the risks associated with the study participation or investigational product administration or may interfere with the interpretation of the results

9 Intervention

9.1 Prescreening

The patients with recurrent glioblastoma seen in outpatient departments of TMH or ACTREC will be considered for this study and screened for same.

9.2 Screening

Patients will be screened post consenting. Each referred patient will be assessed on the basis of study eligibility criteria for this study. Investigations mentioned under baseline assessment would be performed at the time of screening if necessary for eligibility assessment.

9.3 Baseline assessment

Patients would undergo following assessments at baseline

1. History and physical examination
2. Documentation of ECOG PS
3. Blood investigations (If patient already has blood test within 21 days at baseline assessment and has not received any chemotherapy within last 6 weeks then there is no need for repetition of blood investigations.)
    a. Complete blood hemogram
    b. Renal function test inclusive of serum urea and serum creatinine
    c. Liver function test inclusive of serum SGOT. SGPT, T Bilirubin and alkaline phosphatase
    d. Serum serology (HIV, HBsag and HCV)
4. MRI brain (Plain, contrast, spectroscopy and diffusion) : (If patient already has MRI within 21 days at baseline assessment and has not received any chemotherapy within last 6 weeks then there is no need for repetition of MRI.)
5. ECG

9.4 Phase 1

9.4.1 Summary

Patients would be allocated for phase 1 study till the MTD of mebendazole in each arm is discovered. The enrollment in arm A1, B1 & C1 from glioblastoma cohort the eligibility for re-irradiation will be checked. The opinion of joint clinic would be taken for consideration of eligibility for re-irradiation. If patient is eligible then he/she gets in cohort C1. If non eligible then will be randomly assigned 1:1 to either arm A1 or arm B1 in phase I.
3 patients at each dose level would be recruited in each arm. DLT would be captured over the period of radiation in arm A1, over 6 weeks of cycle 1 in arm B1 and over 4 weeks of cycle 1 in arm C1. Only the first cycle would be considered for DLT assessments.

9.4.2 Definitions used for outcome analysis in Phase 1

9.4.2.1 Maximum tolerable dose (MTD)

This is the dose of mebendazole which is associated with 2 or more DLT.

9.4.2.2 Dose limiting toxicity (DLT)

Dose limiting toxicity are grade 3-4 life threatening adverse events related to the combination of investigational drug and other chemotherapeutic agent or radiation which occur during the assigned period of the protocol.

9.4.2.3 Adverse events considered as DLT

The following adverse events are considered as DLT

1. Neutropenia grade 4 or above (CTCAE version 4.03 criteria)
2. Thrombocytopenia grade 4 or above (CTCAE version 4.03 criteria)
3. Rise in SGPT or SGOT of grade 3 and persisting for 1 week (CTCAE version 4.03 criteria)
4. Rise in SGPT or SGOT grade 4 or above (CTCAE version 4.03 criteria)

9.4.2.4 Recording of DLT

Adverse events during the below mentioned period will be considered as DLT and would aid in identifying the MTD. DLT would be captured over the period of radiation in arm A1, over 6 weeks of cycle 1 in arm B1 and over 4 weeks of cycle 1 in arm C1. Only the first cycle would be considered for DLT assessments. In case the disease progress before occurrence of DLT in cycle 1 then such cycle would be considered for DLT assessment and it would be labelled as having no DLT.

9.4.3 Dose levels

Escalating dose of oral mebendazole given daily on D1

1. Dose Level 0: 500 mg TDS
2. Dose Level I: 600 mg TDS
3. Dose Level II: 700 mg TDS
4. Dose Level III: 800 mg TDS
5. Dose Level IV: 1000 mg TDS

9.4.4 Schema of phase 1

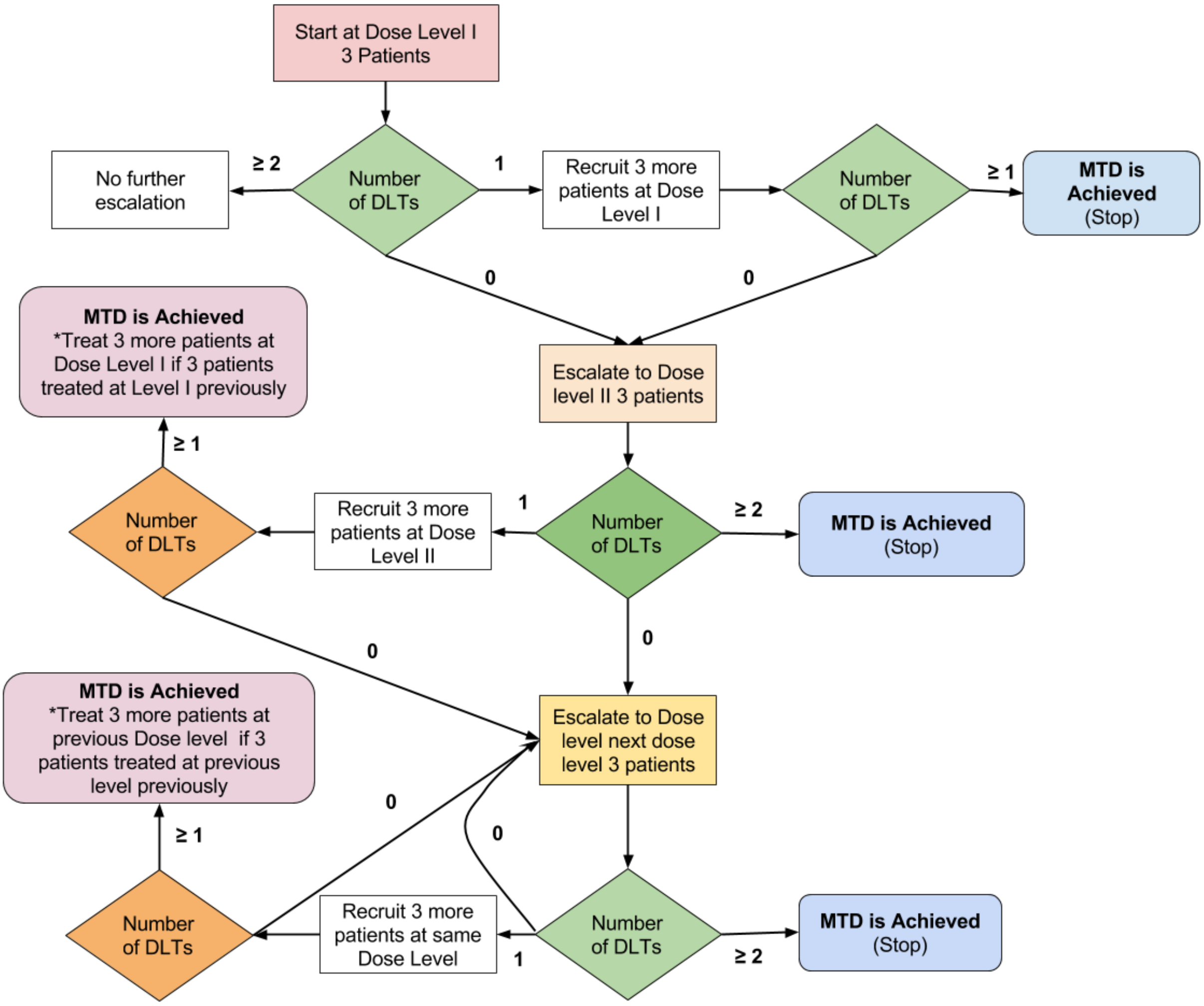

Figure 3: Schema of phase 1.

Number of Patients with DLT at a Given Dose Level	Escalation Decision Rule
0 out of 3	Enter 3 patients at Dose Level + 1 (next dose level)
1 out of 3	Enter 3 more patients at this Dose Level again.<br>1. If 0 of these 3 patients experience DLT then proceed to Dose Level + 1 (next dose level)<br>2. If 1 or more of this group experience DLT then stop. This dose is the MTD.<br>a. 3 more patients to be entered at the Dose Level - 1 (previous dose level) if only 3 patients were treated at that dose level
2 out of 3	MTD is considered to be achieved.

	1. 3 more patients to be entered at the Dose Level - 1 (previous dose level) if only 3 patients were treated at that dose level

Table 1: Table detailing the dose escalation rules.

3 patients will be enrolled at each dose level cohort in each arm. Post completion of a dose level cohort. The data would be analysed for DLT. The decision rules for further enrollment are shown in figure 3 and table 1.

9.4.5 Selection of Arm

9.4.5.1 Glioblastoma histology

The enrollment in arm A1, B1 & C1 from glioblastoma cohort the eligibility for re-irradiation will be checked. The opinion of joint clinic would be taken for consideration of eligibility for re-irradiation. If patient is eligible then he/she gets in cohort C1. If non eligible then will be randomly assigned to either arm A1 or arm B1 in phase I. If MTD is reached in any arm earlier than the other then patients would be allotted to the arms in which MTD is not reached.

9.4.6 Administration of drug

Mebendazole in each arm will be administered as 100 mg tablet. The tablets would be chewed with food by the patients. The number of tablets at each dose level per dosing will be chewed either together or one by one. The drug will be administered daily. The duration would be

1. Arm A1: From start of radiation till end
2. Arm B1: From day 1 to day 42 of each cycle
3. Arm C1: From day 1 to day 28 of each cycle.

9.4.7 Companion drug in Arm A1, B1 & C1

9.4.7.1 Arm A1

9.4.7.1.1 Radiation

Curative radiation would be administered. The planning, contour delineation, dosing and administration of radiation would be done in accordance with institutional standards.

9.4.7.1.2 Temozolomide

Temozolomide would be administered in a dose of 75 mg/m^2 once daily from start of radiation till its end. It would be administered empty stomach with concurrent 5HT3 inhibitor. The dose would be rounded off to the nearest dose possible with 100 mg tablet. The sequence of administration of drugs in arm A1 would be

Time	Drug
Early morning empty stomach	Tablet pantoprazole 40 mg
Breakfast	Mebendazole tablets would be chewed with breakfast
1 ½ hours post breakfast	Tablet granisetron 1 mg or 8 mg ondansetron
2 hours post breakfast	Tablet temozolomide (75 mg/m^2)
Lunch	Mebendazole tablets would be chewed with lunch
Dinner	Mebendazole tablets would be chewed with dinner

Table 2: table showing the sequence of drug administration in arm A1

9.4.7.1.3 Duration of treatment

During the whole course of radiation.

9.4.7.1.4 Compliance criteria

The temozolomide and mebendazole should be started within 3 days of start of radiation and no more than 5 doses of these tablets should be missed by the patient. Tablets missed due to adverse events won't be considered as noncompliance. The compliance would be documented at each visit. Noncompliance in any drug beyond the limits mentioned above will lead to disqualification of patient from the study. This cycle won't be considered for DLT and would be replaced.

9.4.7.1.5 Adverse events recording

The adverse events would be recorded on every week of RT. The following adverse events recorded

1. Hematological adverse events
    a. Anemia
    b. Neutropenia
    c. Thrombocytopenia
    d. Febrile neutropenia
2. Renal dysfunction
    a. Rise in serum creatinine
3. Liver dysfunction
    a. Rise in SGOT and or SGPT
    b. Rise in T bilirubin
4. Clinical events
    a. Nausea
    b. Vomiting
    c. Mucositis

    d. Diarrhea
5. Other events: any other events of clinical significance if occurs would be recorded
    a. Any other clinical event

9.4.7.2 Arm B1

9.4.7.2.1 CCNU

CCNU would be administered in a dose of 110 mg/m^2 once on day 1 of 42 days cycle. It would be administered with concurrent 5HT3 inhibitor. The dose would be rounded off to the nearest dose possible with 40 mg capsule. The sequence of administration of drugs in arm B1 would be

Time	Drug
Early morning empty stomach	Tablet pantoprazole 40 mg
Breakfast	Mebendazole tablets would be chewed with breakfast
1 ½ hours post breakfast	Tablet granisetron 1 mg or ondansetron 8 mg
2 hours post breakfast	Capsule CCNU (110 mg/m^2)
Lunch	Mebendazole tablets would be chewed with lunch
Dinner	Mebendazole tablets would be chewed with dinner

Table 3 : Table showing the sequence of drug administration in arm B1

9.4.7.2.2 Duration of treatment

CCNU: day 1 of each cycle
Mebendazole: daily
Each cycle is of 42 days. Maximum 6 cycles would be administered.

9.4.7.2.3 Compliance criteria

The CCNU tablet has to be taken on day 1. Mebendazole should be started within 3 days of start of CCNU and no more than 7 doses of these tablets should be missed by the patient. Tablets missed due to adverse events won't be considered as noncompliance. The compliance would be documented at each visit. Noncompliance in any drug beyond the limits mentioned above will lead to disqualification of patient from the study. This cycle won't be considered for DLT and would be replaced.

9.4.7.2.4 Adverse events recording

The adverse events would be recorded on day 14, day 28 and day 42 of cycle 1. The following adverse events recorded

1. Hematological adverse events
    a. Anemia
    b. Neutropenia
    c. Thrombocytopenia
    d. Febrile neutropenia
2. Renal dysfunction
    a. Rise in serum creatinine
3. Liver dysfunction
    a. Rise in SGOT and or SGPT
    b. Rise in T bilirubin
4. Clinical events
    a. Nausea
    b. Vomiting
    c. Mucositis
    d. Diarrhea
5. Other events: any other events of clinical significance if occurs would be recorded
    a. Any other clinical event

9.4.7.3 Arm C1

9.4.7.3.1 Temozolomide

Temozolomide would be administered in a dose of 200 mg/m^2 once daily from day 1 to day 5. It would be administered empty stomach with concurrent 5HT3 inhibitor. The dose would be rounded off to the nearest dose possible with 100 mg tablet. The sequence of administration of drugs in arm C1 would be

Time	Drug
Early morning empty stomach	Tablet pantoprazole 40 mg
Breakfast	Mebendazole tablets would be chewed with breakfast
1 ½ hours post breakfast	Tablet granisetron 1 mg or ondansetron 8 mg
2 hours post breakfast	Tablet temozolomide (200 mg/m^2)
Lunch	Mebendazole tablets would be chewed with lunch
Dinner	Mebendazole tablets would be chewed with dinner

Table 4: Table showing the sequence of drug administration in arm C1

9.4.7.3.2 Duration of treatment

Temozolomide: day 1-day 5 of each cycle
Mebendazole: daily
Each cycle is of 28 days. Maximum 12 cycles would be administered.

9.4.7.3.3 Compliance criteria

The temozolomide would be started on day 1 and no more than 1 dose can be missed. Mebendazole would be started within 3 days of start of temozolomide and no more than 5 doses of these tablets would be missed by the patient. Tablets missed due to adverse events won't be considered as noncompliance. The compliance would be documented at each visit. Non compliance in any drug beyond the limits mentioned above will lead to disqualification of patient from the study. This cycle won't be considered for DLT and would be replaced.

9.4.7.3.4 Adverse events recording

The adverse events would be recorded on day 7, day 14 and day 28 of cycle 1. The following adverse events recorded

1. Hematological adverse events
    a. Anemia
    b. Neutropenia
    c. Thrombocytopenia
    d. Febrile neutropenia
2. Renal dysfunction
    a. Rise in serum creatinine
3. Liver dysfunction
    a. Rise in SGOT and or SGPT
    b. Rise in T bilirubin
4. Clinical events
    a. Nausea
    b. Vomiting
    c. Mucositis
    d. Diarrhea
5. Other events: any other events of clinical significance if occurs would be recorded
    a. Any other clinical event

9.4.8 Criteria for starting first cycle

The following requirements need to be satisfied before starting the first cycle

a. Hemoglobin ≥ 8 g/dl
b. Leukocytes ≥2,000/mcL or ANC ≥ 1500/mcL
c. Platelets ≥100,000/mcL
d. Total bilirubin < 1.5 × institutional upper limit of normal
e. AST(SGOT)/ALT(SGPT) ≤2.5 × institutional upper limit of normal

9.4.9 Dose modifications

9.4.9.1 Mebendazole

No known modifications for hepatic dysfunction, renal dysfunction or myelosuppression

9.4.9.2 Temozolomide

ANC (10^9/L)		Platelet(10^9/L)		Non hematological toxicity#	Dose for next cycle
<1	or	<50	or	Grade 3	Reduce by dose level 1*
-	or	-	or	Grade 4 or Recurrent Grade 3 or pneumonitis or severe rash	Discontinue
				Hepatotoxicity	Assess risk/benefit before continuing treatment
				Hepatitis B	Discontinue if active disease or reactivation
				Serum creatinine clearance below 40 ml/min	To be used with caution

Table 5: Dose modification for temozolomide. Dose levels are 200, 150 and 100 mg/m2. # except for alopecia, nausea, vomiting. * Discontinue if < 100 mg/m2

9.4.9.3 CCNU

9.4.9.3.1 Dose modification for hepatic dysfunction

None

9.4.9.3.2 Dose modification for renal dysfunction

Creatinine clearance	Lomustine dose
> 50 ml/min	100%
10-50 ml/min	75%
< 10 ml/min	50%

Table 6: Dose modification of CCNU in case of renal dysfunction

9.4.9.3.3 Dose modification for myelosuppression

The dose modifications will be done taking into account the myelosuppression in previous cycle.
1. The dose will be reduced by 20-25% in case of decrease in ANC between 1.5-1.0X10^9/L or a decrease in platelet count between 80-100 X 10^9/L.
2. The dose will be reduced by 40-50% in case of decrease in ANC below 1.0X10^9/L or a decrease in platelet count below 80 X 10^9/L.

9.4.10 Accompanying medications

Contraindicated<br>1. Cimetidine CI with mebendazole and CCNU
Caution<br>1. Valproic acid when temozolomide is used

Table 7: Prohibited medications

9.4.11 Criteria for discontinuing protocol

Participant’s choice

1. Participant demonstrates an inability or unwillingness to comply with the oral medication regimen and/or documentation requirements
2. Participant decides to withdraw from the study
3. General or specific changes in the participant's condition render the participant unacceptable for further treatment in the opinion of the treating investigator.
4. Inter-current illness that prevents further administration of treatment
5. Delay of treatment for 14 days due to treatment-related adverse events. For delays >14 days due to due to reasons other than treatment-related adverse events. The treatment would be continued. For delay > 21 days due to due to reasons other than treatment-related adverse events. The treatment would be discontinued.
6. Incompliance with medications as explained in section 10.4.7.1.3, 10.4.7.2.3 or 10.4.7.3.3 whichever is applicable.
7. Occurrence of an exclusion criterion affecting patient safety, e.g., pregnancy or psychiatric illness.

9.4.12 Post protocol care

1. In case of non-progression of disease post Cycle 1 in arm B1 & C1 and post completion of radiation in arm A1:
    a. The patient in arm A1 would receive further treatment in accordance with institutional standards.

    b. The patient in arm B1 & C1 would receive the respective companion drugs and mebendazole would be administered in same dose as in cycle 1 till progression.
2. In case of progression of disease
    a. Progression during cycle 1 in arm B1 & C1 or during RT in arm A1: In case the disease progress before occurrence of DLT in cycle 1 or during RT in arm A1 then such cycle would be considered for DLT assessment and it would be labelled as having no DLT. The further treatment would be in accordance with institutional standards.
    b. Progression post cycle 1 in arm B1 & C1 or post RT in arm A1: The further treatment would be in accordance with institutional standards.

9.5 Phase 2

9.5.1 Summary of phase 2

Phase 2 will start post completion of phase 1. In phase 2 there would be 3 arms. Each arm would allocate 44 patients. These patients would be treated with mebendazole at MTD (for the respective companion drug) with the companion drug. The toxicity, adverse events and overall survival would be documented.

9.5.2 Selection of Arm

9.5.2.1 Glioblastoma histology

The enrollment in arm A2, B2 & C2 from glioblastoma cohort, the eligibility for re-irradiation will be checked. The opinion of joint clinic would be taken for consideration of eligibility for re-irradiation. If patient is eligible then he/she gets in cohort C2. If non eligible then will be randomly assigned to either arm A2 or arm B2 in phase II.

9.5.3 Administration of drug

9.5.3.1 Mebendazole

Mebendazole in each arm will be administered at MTD as 100 mg tablet. The tablets would be chewed with food by the patients. The number of tablets at each dose level per dosing will be chewed either together or one by one. The drug will be administered daily . The duration would be

1. Arm A2: From start of radiation till end
2. Arm B2: From day 1 to day 42 of each cycle
3. Arm C2: From day 1 to day 28 of each cycle.

9.5.4 Companion drug in Arm A2, B2, C2

9.5.4.1 Arm A2

9.5.4.1.1 Radiation

The details of radiation are same as in arm A1 section

9.5.4.1.2 Temozolomide

Temozolomide would be administered in a dose of 75 mg/m2 once daily from start of radiation till its end. It would be administered empty stomach with concurrent 5HT3 inhibitor The dose would be rounded off to the nearest dose possible with 100 mg tablet. The sequence of administration of drugs in arm A1 would be

Time	Drug
Early morning empty stomach	Tablet pantoprazole 40 mg
Breakfast	Mebendazole tablets would be chewed with breakfast
1 ½ hours post breakfast	Tablet granisetron 1 mg or ondansetron 8 mg
2 hours post breakfast	Tablet temozolomide (75 mg/m^2)
Lunch	Mebendazole tablets would be chewed with lunch
Dinner	Mebendazole tablets would be chewed with dinner

Table 8: Table showing the sequence of drug administration in arm A2

9.5.4.1.3 Duration of treatment

During the whole course of radiation.

9.5.4.1.4 Compliance criteria

The temozolomide and mebendazole should be started within 3 days of start of radiation and no more than 5 doses of these tablets should be missed by the patient. Tablets missed due to adverse events won't be considered as noncompliance. The compliance would be documented at each visit.

9.5.4.1.5 Adverse events recording

The adverse events would be recorded every week till radiation is ongoing. The following adverse events recorded

1. Hematological adverse events
    a. Anemia

    b. Neutropenia
    c. Thrombocytopenia
    d. Febrile neutropenia
2. Renal dysfunction
    a. Rise in serum creatinine
3. Liver dysfunction
    a. Rise in SGOT and or SGPT
    b. Rise in T bilirubin
4. Clinical events
    a. Nausea
    b. Vomiting
    c. Mucositis
    d. Diarrhea
5. Other events: any other events of clinical significance if occurs would be recorded
    a. Any other clinical event

9.5.4.2 Arm B2

9.5.4.2.1 CCNU

CCNU would be administered in a dose of 110 mg/m2 once on day 1 of 42 days cycle. It would be administered with concurrent 5HT3 inhibitor. The dose would be rounded off to the nearest dose possible with 40 mg capsule. The sequence of administration of drugs in arm B1 would be

Time	Drug
Early morning empty stomach	Tablet pantoprazole 40 mg
Breakfast	Mebendazole tablets would be chewed with breakfast
1 ½ hours post breakfast	Tablet granisetron 1 mg or ondansetron 8 mg
2 hours post breakfast	Capsule CCNU (110 mg/m^2)
Lunch	Mebendazole tablets would be chewed with lunch
Dinner	Mebendazole tablets would be chewed with dinner

Table 9: Table showing the sequence of drug administration in arm B2

9.5.4.2.2 Duration of treatment

CCNU: day 1 of each cycle
Mebendazole: daily
Each cycle is of 42 days. Maximum 6 cycles would be administered.

9.5.4.2.3 Compliance criteria

The CCNU tablet has to be taken on day 1. Mebendazole should be started within 3 days of start of CCNU and no more than 7 doses of these tablets should be missed by the patient. Tablets missed due to adverse events won't be considered as noncompliance. The compliance would be documented at each visit.

9.5.4.2.4 Adverse events recording

The adverse events would be recorded on day 42 of each cycle. The following adverse events recorded

1. Hematological adverse events
   a. Anemia
   b. Neutropenia
   c. Thrombocytopenia
   d. Febrile neutropenia
2. Renal dysfunction
   a. Rise in serum creatinine
3. Liver dysfunction
   a. Rise in SGOT and or SGPT
   b. Rise in T bilirubin
4. Clinical events
   a. Nausea
   b. Vomiting
   c. Mucositis
   d. Diarrhea
5. Other events: any other events of clinical significance if occurs would be recorded
   a. Any other clinical event

9.5.4.3 Arm C2

9.5.4.3.1 Temozolomide

Temozolomide would be administered in a dose of 200 mg/m^2 once daily from day 1 to day 5. It would be administered empty stomach with concurrent 5 HT3 inhibitor. The dose would be rounded off to the nearest dose possible with 100 mg tablet. The sequence of administration of drugs in arm C1 would be

Time	Drug
Early morning empty stomach	Tablet pantoprazole 40 mg
Breakfast	Mebendazole tablets would be chewed with breakfast
1 ½ hours post breakfast	Tablet granisetron 1 mg or ondansetron 8 mg

2 hours post breakfast	Tablet temozolomide (200 mg/m^2)
Lunch	Mebendazole tablets would be chewed with lunch
Dinner	Mebendazole tablets would be chewed with dinner

Table 10: Table showing the sequence of drug administration in arm C2

9.5.4.3.2 Duration of treatment

Temozolomide: day 1-day 5 of each cycle
Mebendazole: daily
Each cycle is of 28 days. Maximum 12 cycles would be administered.

9.5.4.3.3 Compliance criteria

The temozolomide would be started on day 1 and no more than 1 dose can be missed. Mebendazole would be started within 3 days of start of temozolomide and no more than 5 doses of these tablets would be missed by the patient. Tablets missed due to adverse events won't be considered as noncompliance. The compliance would be documented at each visit.

9.5.4.3.4 Adverse events recording

The adverse events would be recorded on day 28 of cycle 1. The following adverse events recorded

1. Hematological adverse events
    a. Anemia
    b. Neutropenia
    c. Thrombocytopenia
    d. Febrile neutropenia
2. Renal dysfunction
    a. Rise in serum creatinine
3. Liver dysfunction
    a. Rise in SGOT and or SGPT
    b. Rise in T bilirubin
4. Clinical events
    a. Nausea
    b. Vomiting
    c. Mucositis
    d. Diarrhea
5. Other events: any other events of clinical significance if occurs would be recorded
    a. Any other clinical event

9.5.5 Criteria for starting first cycle

The following requirements need to be satisfied before starting the first cycle

1. Hemoglobin ≥ 8 g/dl
2. Leukocytes ≥2,000/mcL or ANC ≥ 1500/mcL
3. Platelets ≥100,000/mcL
4. Total bilirubin < 1.5 × institutional upper limit of normal
5. AST(SGOT)/ALT(SGPT) ≤2.5 × institutional upper limit of normal

9.5.6 Criteria for starting second cycle (Arm B2, C2) or continuing TMZ in Arm A2

1. Hemoglobin ≥ 8 g/dl
2. Leukocytes ≥2,000/mcL or ANC ≥ 1500/mcL
3. Platelets ≥100,000/mcL
4. Total bilirubin < 1.5 × institutional upper limit of normal
5. AST(SGOT)/ALT(SGPT) ≤2.5 × institutional upper limit of normal
6. All non-hematological toxicities should have resolved to grade 2 or less

9.5.7 Dose modifications

Please see the section 10.4.9

9.5.8 Accompanying medications

Please see the section 10.4.10

9.5.9 Documentation of response and quality of life

9.5.9.1 Response assessment

Response assessment will be done in accordance with institutional standards. MRI brain +/- spine (Plain, contrast, perfusion and spectroscopy) will be used for assessment.

1. Arm A 2: It will be done post 1 month (+/- 10 days) of completion of radiation. The further response assessment will be in accordance with institutional practice.
2. Arm B2: It will be done post 3 cycles and 6 cycles of CCNU or earlier if clinically indicated. The further response assessment will be in accordance with institutional practice.
3. Arm C2: It will be done post 6 cycles and 12 cycles of temozolomide or earlier if clinically indicated. The further response assessment will be in accordance with institutional practice.

In case of clinical or radiological progression or decrement in PS the treatment arm will be discontinued.

9.5.9.2 Quality of life assessment

Quality of life assessment in each arm will be done at baseline, 3 months (+/- 15 days) and at 6 months (+/- 1 month). The EORTC QLQ C-30 with Brain (QLQ-BN20) module would be administered to the patients. Patients assessed with cognitive decline would be exempted.

9.5.10 Criteria for discontinuing protocol

1. Participant’s choice
2. Participant demonstrates an inability or unwillingness to comply with the oral medication regimen and/or documentation requirements
3. Participant decides to withdraw from the study
4. General or specific changes in the participant's condition render the participant unacceptable for further treatment in the opinion of the treating investigator.
5. Intercurrent illness that prevents further administration of treatment
6. Delay of treatment for 14 days due to treatment-related adverse events. For delays >14 days due to reasons other than treatment-related adverse events. The treatment would be continued. For delay > 21 days due to reasons other than treatment-related adverse events. The treatment would be discontinued.
7. Occurrence of an exclusion criterion affecting patient safety, e.g., pregnancy or psychiatric illness.

9.5.11 Post protocol care

In case of progression or discontinuation of treatment on any arm: The further treatment would be in accordance with institutional standards.

10 Outcomes

10.1 Phase 1 objective

To determine MTD of mebendazole in each arm.
Please refer to Table 1 and figure 3 for outcome analysis for determination of MTD.

10.2 Phase 2 objective

10.2.1 Primary outcome for recurrent glioblastoma cohorts: 9-month OS

9-month overall survival in arm A2, B2 and C2: The overall survival will be calculated from date of enrollment in the study till the date of death. Patients who have lost to follow up will be considered as died at the known documented last follow up. Patients who are alive at the time of analysis will be censored. Kaplan Meier method would be used for estimation of overall survival.

10.2.2 Secondary outcomes

1. Progression free survival. Progression free survival in each arm will be calculated from date of enrollment in the study till the date of progression. Patients who have lost to follow up will be considered as progressed at the known documented last follow up. Patients who have not progressed at the time of analysis will be censored. Kaplan Meier method would be used for estimation of median progression free survival.
2. Quality of life : The improvement in qol at 3 months and 6 months in each arm would be seen.
3. Toxicity : The incidence of grade 3-4 adverse events during treatment in each arm in accordance with CTCAE version 4.03 will be recorded. The incidence of maximum grade of adverse event during the whole course of treatment in each arm will be reported. The reporting will be done in terms of percentage with 95% CI wherever appropriate.

11 Participant timeline

11.1 Phase 1

	Baseline	Every week till RT continues						
Hemogram	X	X	X	X	X	X	X	X
Biochemistry	X	X	X	X	X	X	X	X
Adverse events	X	X	X	X	X	X	X	X

Timeline for Phase 1-Arm A1 : DLT recording

	Baseline	Day 7	Day 14	Day 28	Day 42
Hemogram	X	X	X	X	X
Biochemistry	X	X	X	X	X

Adverse events	X	X	X	X	X

Timeline for Phase 1-Arm B1 : DLT recording

	Baseline	Day 7	Day 14	Day 28
Hemogram	X	X	X	X
Biochemistry	X	X	X	X
Adverse events	X	X	X	X

Timeline for Phase 1-Arm C1 : DLT recording

11.2 Phase 2

	Baseline	Every week till RT continues	1 month post RT	2 monthly intervals till progression for 1 year
Hemogram	X	X	X	X
Biochemistry	X	X	X	X
Adverse events	X	X	X	X
MRI	X	-	X	X
QOL	X	-	X	X-at 6 months Visit

Timeline for Phase 2-Arm A2

	Baseline	Day 1 of C2 to C3	Day 1 C4	Day 1 of C5 to C6	1 month post Cycle 6	2 monthly intervals till progression for 1 year
Hemogram	X	X	X	X	X	X
Biochemistry	X	X	X	X	X	X
Adverse	X	X	X	X	X	X

events						
MRI	X	-	X	-	X	-
QOL	X	X-C3D1	-	X C5D1	-	-

Timeline for Phase 2-Arm B2

	Baseline	Day 1 of C2 to C6	Day 1 C7	Day 1 of C8 to C12	1 month post Cycle 12
Hemogram	X	X	X	X	X
Biochemistry	X	X	X	X	X
Adverse events	X	X	X	X	X
MRI	X	-	X	-	X
QOL	X	X-C4D1	X C7D1	-	-

Timeline for Phase 2-Arm C2

12 Sample size

Phase 1 : No sample size estimation. Please see phase 1 schema. A minimum of 9 patients and a maximum of 63 patients would be required.

Phase 2 : An A'Hern one-stage design was used to calculate the sample size needed for each of the treatment arms. With P0 set at 35% (ie, a true overall survival at 9 months of 35% was judged to be too low to warrant further investigation in arm A2, B2 & C2 and P1 set at 55% (ie, a true overall survival at 9 months of 55% was judged sufficient to warrant further studies with the corresponding regimen in arms A2, B2 & C2), and with α of 0·10 and β of 0·10, a total of 44 patients were needed per group. On the basis of these assumptions, if 20 of 44 or more patients were still alive at 9 months in groups (A2, B2 or C2), we would be able to conclude that that specific treatment warrants further investigation in clinical studies.

13 Data collection method and management

The data regarding the patients and the proformas would be collected on pre designed electronic forms. These electronic data would be accessed only by the trials PI, Co-PI, trial coordinator and nurse. Missing data won't be statistically adjusted.

14 Data monitoring

The data would be monitored monthly by the PI for completion. An independent monitoring committee from DSMC will review the trial conduct and data yearly.

15 Harms

The life threatening complication rate with mebendazole are below 1%. Long term safety data with 50 mg/kg per day dose given for 24 months at least is available. Hence chances of harm due to the drug mebendazole seems slim.

16 Auditing

The data will be monitored by the PI and the Co-Pi independently. At least one-third case records will be doubly checked every months.

17 Ethics approval

This protocol and the template informed consent forms contained in Appendix will be reviewed and approved by the institutional IRB with respect to scientific content and compliance with applicable research and human subjects regulations. The protocol, participant education and recruitment materials, and other requested documents—and any subsequent modifications —also will be reviewed and approved by the IRB. Subsequent to initial review and approval, the IRB will review the protocol at least annually. The Investigator will make safety and progress reports to the IRB at 12 monthly intervals and post study termination or completion. These reports will include the total number of participants enrolled and summaries of each DSMB [data safety and monitoring board] review of safety and/or efficacy.

18 Protocol amendments

Any modifications to the protocol which may impact on the conduct of the study, potential benefit of the patient or may affect patient safety, including changes of study objectives, study design, patient population, sample sizes, study procedures, or significant administrative aspects will require a formal amendment to the protocol. Any and all such amendments will be communicated to the institutional IRB for review and approval. Administrative changes of the protocol are minor corrections and/or clarifications that have no effect on the way the study is to be conducted. These may be communicated to the IRB at the investigator's' discretion.

19 Consent or assent

Patients will be given the patient information sheet by the trial investigators / nurses. The purpose and reasons behind the study will be communicated to the patient. All patients will be provided with a copy of the written informed consent as well as the patient information sheet. Consent will be recorded on paper as per institutional IRB guidelines.

20 Confidentiality

All study-related information will be stored securely at the study site. All participant information will be stored in locked file cabinets in areas with limited access. All records that contain names or other personal identifiers, such as locator forms and informed consent forms, will be stored separately from study records identified by code number. All local databases will be secured with password-protected access systems. Forms, lists, log books, appointment books, and any other listings that link participant ID numbers to other identifying information will be stored in a separate, locked file in an area with limited access.

21 Declaration of interests

Both PIs and Co-PIs have no financial conflicts of interest.

22 Access to data

The Principal Investigators and Co-PI will be given access to the cleaned data sets. Project data sets will be housed on the project specific database created for the study, and all data sets will be password protected.

23 Dissemination policy

The trial results will be submitted for publication in international peer reviewed journals and presented at national/international conferences. No publication restrictions will be imposed by trial sponsors. The principal investigators will take the roles of the corresponding author and first author positions as decided mutually. Authorship criteria for other publications stemming from the study will be decided as per the relative contributions and existing institutional guidelines. The contribution of all investigators will be acknowledged in such manuscripts if they are not eligible for authorship. In addition the grant giving organizations will also be acknowledged in all publications.

24 References

1 Stupp R, Mason WP, van den Bent MJ, *et al.* Radiotherapy plus concomitant and adjuvant temozolomide for glioblastoma. *N Engl J Med* 2005; **352**: 987–96.

2 Stupp R, Hegi ME, Mason WP, *et al.* Effects of radiotherapy with concomitant and adjuvant temozolomide versus radiotherapy alone on survival in glioblastoma in a randomised phase III study: 5-year analysis of the EORTC-NCIC trial. *Lancet Oncol* 2009; **10**: 459–66.

3 Wick W, Platten M, Meisner C, *et al.* Temozolomide chemotherapy alone versus radiotherapy alone for malignant astrocytoma in the elderly: the NOA-08 randomised, phase 3 trial. *Lancet Oncol* 2012; **13**: 707–15.

4 Kirkpatrick JP, Sampson JH. Recurrent malignant gliomas. *Semin Radiat Oncol* 2014; **24**: 289–98.

5 Souhami L, Seiferheld W, Brachman D, *et al.* Randomized comparison of stereotactic radiosurgery followed by conventional radiotherapy with carmustine to conventional radiotherapy with carmustine for patients with glioblastoma multiforme: report of Radiation Therapy Oncology Group 93-05 protocol. *Int J Radiat Oncol Biol Phys* 2004; **60**: 853–60.

6 Møller S, Grunnet K, Hansen S, *et al.* A phase II trial with bevacizumab and irinotecan for patients with primary brain tumors and progression after standard therapy. *Acta Oncol* 2012; **51**: 797–804.

7 Taal W, Oosterkamp HM, Walenkamp AME, *et al.* Single-agent bevacizumab or lomustine versus a combination of bevacizumab plus lomustine in patients with recurrent glioblastoma (BELOB trial): a randomised controlled phase 2 trial. *Lancet Oncol* 2014; **15**: 943–53.

8 Wick W, Puduvalli VK, Chamberlain MC, *et al.* Phase III study of enzastaurin compared with lomustine in the treatment of recurrent intracranial glioblastoma. *J Clin Oncol* 2010; **28**: 1168–74.

9 Batchelor TT, Mulholland P, Neyns B, *et al.* Phase III randomized trial comparing the efficacy of cediranib as monotherapy, and in combination with lomustine, versus lomustine alone in patients with recurrent glioblastoma. *J Clin Oncol* 2013; **31**: 3212–8.

10 Brandes AA, Carpentier AF, Kesari S, *et al.* A Phase II randomized study of galunisertib monotherapy or galunisertib plus lomustine compared with lomustine monotherapy in patients with recurrent glioblastoma. *Neuro Oncol* 2016; **18**: 1146–56.

11 Franceschi E, Stupp R, van den Bent MJ, *et al.* EORTC 26083 phase I/II trial of dasatinib in combination with CCNU in patients with recurrent glioblastoma. *Neuro Oncol* 2012; **14**: 1503–10.

12 Galanis E, Anderson SK, Lafky JM, *et al.* Phase II study of bevacizumab in combination with sorafenib in recurrent glioblastoma (N0776): a north central cancer treatment group trial. *Clin Cancer Res* 2013; **19**: 4816–23.

13 Kreisl TN, Smith P, Sul J, *et al.* Continuous daily sunitinib for recurrent glioblastoma. *J Neurooncol* 2013; **111**: 41–8.

14 Kreisl TN, McNeill KA, Sul J, Iwamoto FM, Shih J, Fine HA. A phase I/II trial of vandetanib for patients with recurrent malignant glioma. *Neuro Oncol* 2012; **14**: 1519–26.

15 Balmaceda C, Peereboom D, Pannullo S, *et al.* Multi-institutional phase II study of temozolomide administered twice daily in the treatment of recurrent high-grade gliomas. *Cancer* 2008; **112**: 1139–46.

16 Omuro A, Chan TA, Abrey LE, *et al.* Phase II trial of continuous low-dose temozolomide for patients with recurrent malignant glioma. *Neuro Oncol* 2013; **15**: 242–50.

17 Bai R, Staedtke V, Rudin C, Bunz F, Riggins G. ET-04MEBENDAZOLE IS EFFICACIOUS IN DIVERSE MEDULLOBLASTOMA TUMOR MODELS AND INHIBITS TUMOR ANGIOGENESIS. *Neuro Oncol* 2014; **16**: v79–v79.

18 Riggins G, Bai R, Gallia G. ATNT-21PRECLINICAL AND INITIAL PHASE 1 EXPERIENCE FOR USE OF MEBENDAZOLE AS UP FRONT THERAPY FOR HIGH GRADE GLIOMA. *Neuro Oncol* 2015; **17**: v15–v15.

19 Microsoft Word - VER11192014CPM. https://www.janssen.com/canada/sites/www_janssen_com_canada/files/product/pdf/ver11192014cpm.pdf.

20 Bai R-Y, Staedtke V, Wanjiku T, *et al.* Brain Penetration and Efficacy of Different Mebendazole Polymorphs in a Mouse Brain Tumor Model. *Clin Cancer Res* 2015; **21**: 3462–70.

21 Reuter S, Jensen B, Buttenschoen K, Kratzer W, Kern P. Benzimidazoles in the treatment of alveolar echinococcosis: a comparative study and review of the literature. *J Antimicrob Chemother* 2000; **46**: 451–6.
